# HSP90AA1 restrains clear cell renal cell carcinoma progression by promoting CADM1 expression and suppressing the PI3K-AKT pathway through interaction with FBXO7

**DOI:** 10.1038/s41420-025-02848-4

**Published:** 2026-01-08

**Authors:** Wuping Yang, Yifan Li, Zhi Li, Chaochao Jiang, Xu Deng, Ding Peng

**Affiliations:** 1 Department of Urology, The First Affiliated Hospital, Zhejiang University School of Medicine, Hangzhou, China; 2 Department of Urology, The First Affiliated Hospital of Hunan Traditional Chinese Medical College, Zhuzhou, China; 3 Department of Urology, Changxing Hospital of Traditional Chinese Medicine, Changxing, China

**Keywords:** Renal cell carcinoma, Tumour-suppressor proteins

## Abstract

Recent studies have shown that heat shock protein 90 alpha family class A member 1 (HSP90AA1) interacts with various tumor-associated proteins, regulates their biological activity and stability, and plays an important role in various tumors. However, the role of HSP90AA1 in clear cell renal cell carcinoma (ccRCC) remains unclear. In the study, GEO and TCGA-KIRC databases were used to analyze the expression pattern and clinical significance of HSP90AA1 in ccRCC; immunohistochemistry and Western blot were used to validate HSP90AA1 expression in ccRCC tissues and cell lines; colony formation assays, EdU and TUNEL methods, cell migration and invasion experiments, and a mouse renal orthotopic xenograft tumor model were used to detect the effects of HSP90AA1 overexpression on the biological function of ccRCC; Co-IP and RNA-seq experiments were utilized to explore the downstream regulatory mechanism of HSP90AA1. Our results showed that HSP90AA1 expression was significantly downregulated in ccRCC, and its reduced expression was associated with tumor metastasis. HSP90AA1 overexpression markedly inhibited the proliferation and metastasis ability of ccRCC cells. HSP90AA1 bound to F-box only protein 7 (FBXO7) and accelerated its protein expression. FBXO7 was expressed at low level in ccRCC, and its decreased expression was closely related to unfavorable pathological features of tumors and poor patient prognosis. FBXO7 overexpression promoted cell adhesion molecule 1 (CADM1) expression and suppressed the PI3K-AKT signaling pathway. Knocking down FBXO7 expression on the basis of HSP90AA1 overexpression significantly reversed the cell phenotype inhibition caused by HSP90AA1 overexpression, downregulated CADM1 expression, and activated the PI3K-AKT signaling pathway. In summary, HSP90AA1 exhibited a low expression pattern in ccRCC, and HSP90AA1 overexpression promoted CADM1 expression and inhibited the PI3K-AKT pathway, thereby suppressing the proliferation and metastasis of ccRCC.

## Introduction

Kidney cancer is one of the most common and deadly tumors of the urinary system, with more than 430,000 new cases and over 150,000 deaths worldwide each year [1]. Renal cell carcinoma (RCC) is a type of cancer that originates from the renal epithelium and accounts for more than 90% of all kidney cancers [2]. Clear cell RCC (ccRCC) is the most common pathological subtype and accounts for over 75% of all RCCs [3]. Although early-stage ccRCC can be surgically removed and has a good prognosis, up to one-third of postoperative patients will progress to metastatic tumors, which are the main cause of patient death [3]. At present, the treatment for advanced ccRCC patients is targeted therapy or a combination of targeted therapy and immunotherapy [4]. However, only a portion of patients demonstrate long-term effectiveness of the treatment [5]. Therefore, it is still necessary to continuously explore new mechanisms of ccRCC occurrence and progression to find new therapeutic targets to promote the development of new drugs and provide more treatment options for patients.

Heat shock protein 90 alpha family class A member 1 (HSP90AA1) is an inducible isoform of the molecular chaperone HSP90 that can assist in the correct folding of specific target proteins. Emerging evidence suggests that HSP90AA1 plays important roles in multiple tumors [6, 7], and it has been shown to interact with various tumor-associated proteins, regulating their biological activity and stability [8]. For example, HSP90AA1 is an unfavorable prognostic factor for hepatocellular carcinoma (HCC) and can lead to tumor development and chemotherapy resistance [9]. HSP90AA1 increases chemotherapy resistance by promoting autophagy and inhibiting apoptosis, and inhibiting HSP90AA1 restores the sensitivity of osteosarcoma cells to chemotherapy in vitro and in vivo [10]. Silencing HSP90AA1 increases the sensitivity of cisplatin (DDP)-resistant head and neck cancer cells to DDP [11]. These studies showed that HSP90AA1 may play a role as an oncogene in tumors. However, other studies have reported that HSP90AA1 can act as a tumor suppressor. For example, the HSP90AA1 gene stabilizes the NME1 protein in breast cancer cells, and its overexpression protects endogenous NME1 protein from degradation, leading to reduced metastasis formation in vitro and in vivo [12]. It can be seen that HSP90AA1 did not show the same inhibitory or promoting effect on different tumors. However, there is currently limited research on HSP90AA1 in RCC, and its expression characteristics, biological function, and mechanism of action remain unclear.

In this study, we aimed to elucidate the expression pattern and clinical significance of HSP90AA1 in ccRCC via analysis of the GEO and TCGA-KIRC public databases and validation in clinical ccRCC samples. Then, cell function experiments and animal experiments were used to clarify the biological function of HSP90AA1 in ccRCC. Finally, western blot, Co-IP and RNA-seq experiments were utilized to explore the potential molecular regulatory mechanism of HSP90AA1.

## Results

### HSP90AA1 expression was downregulated in ccRCC and decreased HSP90AA1 expression was associated with tumor metastasis

**Fig. 1:**
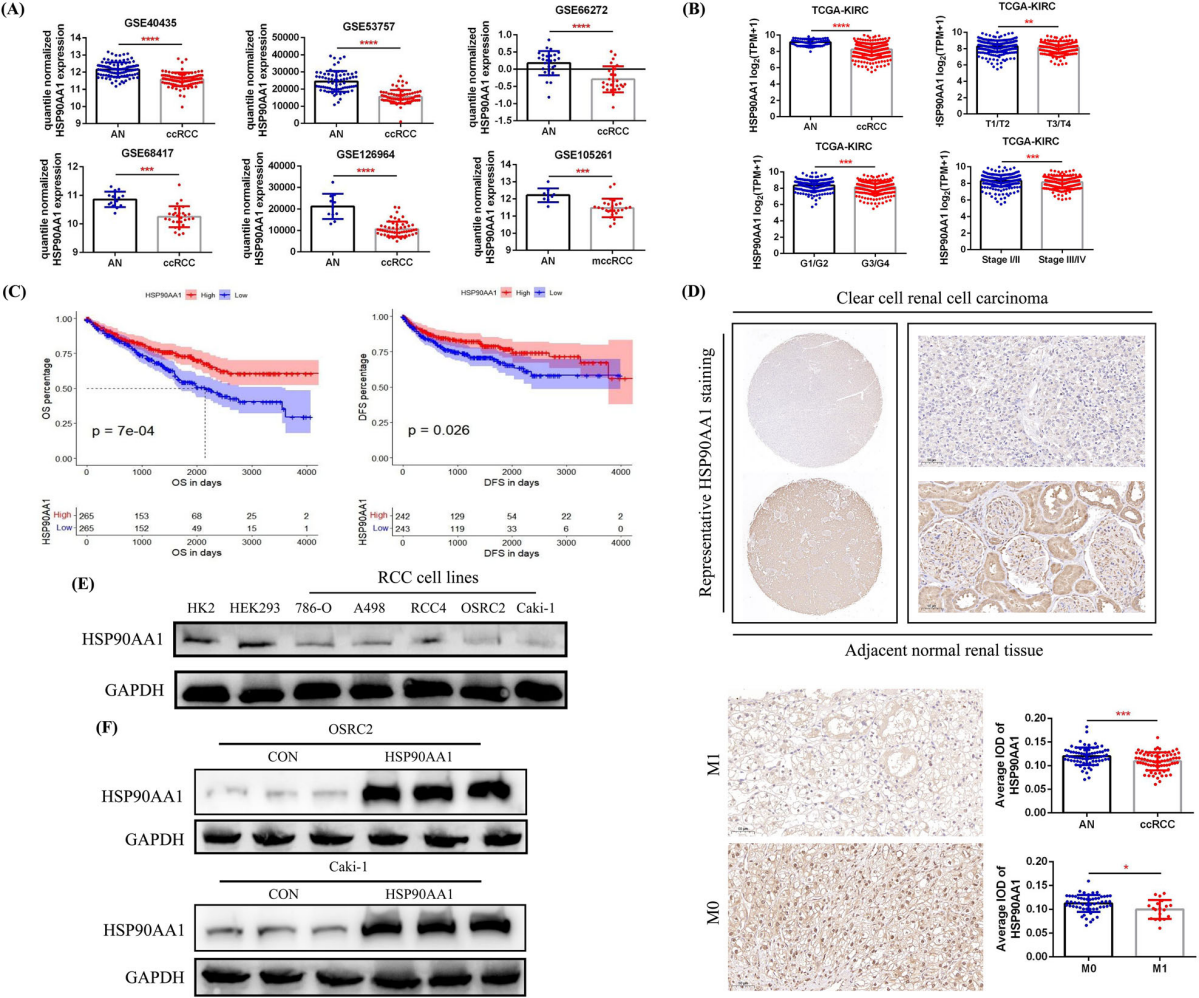
The low expression of HSP90AA1 in ccRCC was verified via the use of public databases and the validation of clinical samples. **A** Comparison of HSP90AA1 expression between ccRCC and AN tissues in 6 GSE datasets. **B** Comparison of HSP90AA1 expression between ccRCC and AN tissues, as well as the expression of HSP90AA1 between tumors of different stages and grades, in the TCGA-KIRC database. AN (*n* = 72, median 9.174), ccRCC (*n* = 539, median 8.271), T1/T2 (*n* = 340, median 8.335), T3/T4 (*n* = 190, median 8.197), G1/G2 (*n* = 241, median 8.360), G3/G4 (*n* = 281, median 8.184), Stage I/II (*n* = 322, median 8.351), Stage III/IV (*n* = 205, median 8.184). **C** According to the median expression of HSP90AA1, tumors in the TCGA-KIRC cohort were divided into high- and low-expression groups for prognostic analysis (OS and DFS). **D** Comparison of HSP90AA1 immunohistochemical staining results between 75 pairs of ccRCC and AN tissues, as well as the expression of HSP90AA1 between M1 and M0 tumors. Scale bar: 50 μm. **E** Comparison of the protein expression of HSP90AA1 between 5 ccRCC cell lines and control cell lines (HK2 and HEK293) via western blot. **F** HSP90AA1 protein was successfully overexpressed in the OSRC2 and Caki-1 cell lines. ^*^P < 0.05, ^**^P < 0.01, ^***^P < 0.001, ^****^P < 0.0001.

To preliminarily clarify the expression characteristics of HSP90AA1 in ccRCC, we used data from 6 GSE datasets (GSE40435, GSE53757, GSE66272, GSE68417, GSE126964, and GSE105261) to compare the mRNA expression of HSP90AA1 between ccRCCs and their AN tissues, and the analysis results confirmed the low expression of HSP90AA1 in ccRCC (Fig. 1A). Using the data from TCGA-KIRC for analysis, the results also revealed that HSP90AA1 expression was significantly reduced in ccRCC and that its low expression was associated with increased tumor stage and grade (T3/T4, G3/G4, and Stage III/IV) (Fig. 1B). Compared with those in the HSP90AA1 low-expression group, the overall survival (OS) and disease-free survival (DFS) of patients in the HSP90AA1 high-expression group were significantly longer (Fig. 1C). To verify the low expression of HSP90AA1 in ccRCC, we used IHC staining to detect the expression of HSP90AA1 in 75 pairs of ccRCC and AN tissues and combined this information with the distant metastasis status of the tumors for further analysis. Our results confirmed the downregulated expression of HSP90AA1 in ccRCC, and its reduced expression was closely related to distant tumor metastasis (Fig. 1D). In addition, we detected HSP90AA1 protein expression in 5 ccRCC cell lines (786-O, A498, RCC4, OSRC2, and Caki-1) via Western blot. Compared with those in the HK2 and HEK293 cell lines, HSP90AA1 expression in these ccRCC cell lines was significantly lower, especially in the metastatic ccRCC cell line Caki-1 (Fig. 1E).

### HSP90AA1 overexpression inhibited ccRCC cell proliferation and metastasis in vitro and in vivo

**Fig. 2:**
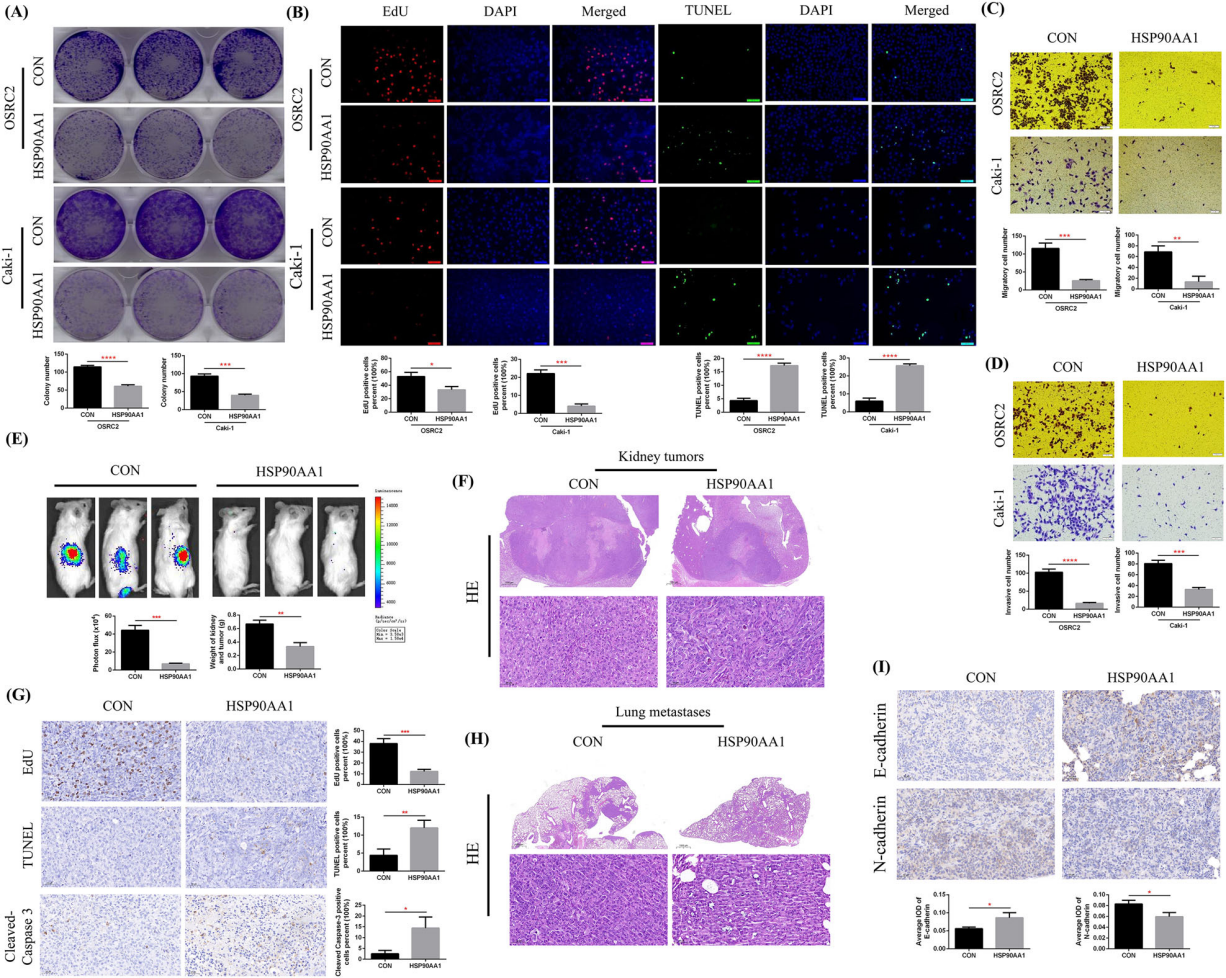
HSP90AA1 overexpression inhibited the proliferation and metastasis of ccRCC cells in vitro and in vivo. **A** Comparison of colony formation numbers between the HSP90AA1-overexpressing group and the control group. **B** Comparison of EdU and TUNEL immunofluorescence results between the HSP90AA1-overexpressing group and the control group. **C**, **D** Comparison of the number of migrating and invading cells between the HSP90AA1-overexpressing group and the control group. **E** Comparison of the bioluminescence imaging results between the renal tumors of the HSP90AA1-overexpressing group and the control group. **F** H&E staining detection of mouse renal tumors. **G** Comparison of EdU, TUNEL, and Cleaved-Caspase 3 expression in the renal tumors of the HSP90AA1-overexpressing group and the control group. **H** H&E staining detection of mouse lung metastases. **I** Comparison of E-cadherin and N-cadherin expression in the lung metastases of the HSP90AA1-overexpressing group and the control group. Scale bar: 50 μm. Repetitions = 3. ^*^P < 0.05, ^**^P < 0.01, ^***^P < 0.001, ^****^P < 0.0001.

After identifying the low expression characteristics of HSP90AA1 in ccRCC, it was essential to clarify the impact of increased HSP90AA1 expression on the tumor phenotype of ccRCC. For this purpose, we overexpressed HSP90AA1 in the OSRC2 and Caki-1 cell lines (Fig. 1F) and detected changes in cell proliferation ability, cell apoptosis, and cell invasiveness in vitro. A plate colony formation assay revealed that the growth ability of ccRCC cells in the HSP90AA1-overexpressing group was significantly weakened compared with that in the control group (Fig. 2A). EdU and TUNEL fluorescence assays revealed that after HSP90AA1 overexpression, ccRCC cell proliferation was significantly decreased and the number of apoptotic cells was markedly increased (Fig. 2B). The results of the cell migration and invasion experiments also revealed that HSP90AA1 overexpression significantly suppressed the invasiveness of ccRCC cells (Fig. 2C, D).

In addition, we constructed a mouse renal orthotopic xenograft tumor growth model to clarify the effect of HSP90AA1 overexpression on ccRCC development in vivo. We orthotopically injected control or HSP90AA1-overexpressing Caki-1 cells into kidney capsules and monitored tumor growth via weekly bioluminescence imaging. The analysis of bioluminescence imaging data confirmed that the growth rate of renal tumors in the HSP90AA1-overexpressing group was significantly lower than that in the control group (Fig. 2E). Then, we used H&E staining to detect the pathological structure of the mouse renal tumors (Fig. 2F). Subsequently, the EdU and TUNEL methods were used to detect cell proliferation and cell apoptosis in renal tumors, respectively. Compared with those in the control group, EdU expression was significantly lower, and TUNEL and Cleaved-Caspase 3 expression was markedly greater in the HSP90AA1-overexpressing group (Fig. 2G). We also used H&E staining to detect the presence and pathological structure of the mouse lung metastases (Fig. 2H). Moreover, we used IHC staining to detect the expression of E-cadherin (a tumor invasion inhibitory factor) and N-cadherin (a tumor invasion initiating factor) in lung metastases. Our results confirmed that E-cadherin expression was significantly increased, whereas N-cadherin expression was markedly reduced in the HSP90AA1-overexpressing group (Fig. 2I).

Taken together, our above cell and animal experimental results fully demonstrated the inhibitory effect of HSP90AA1 overexpression on the proliferation and metastasis of ccRCC cells.

### HSP90AA1 bound to the FBXO7 protein

**Fig. 3:**
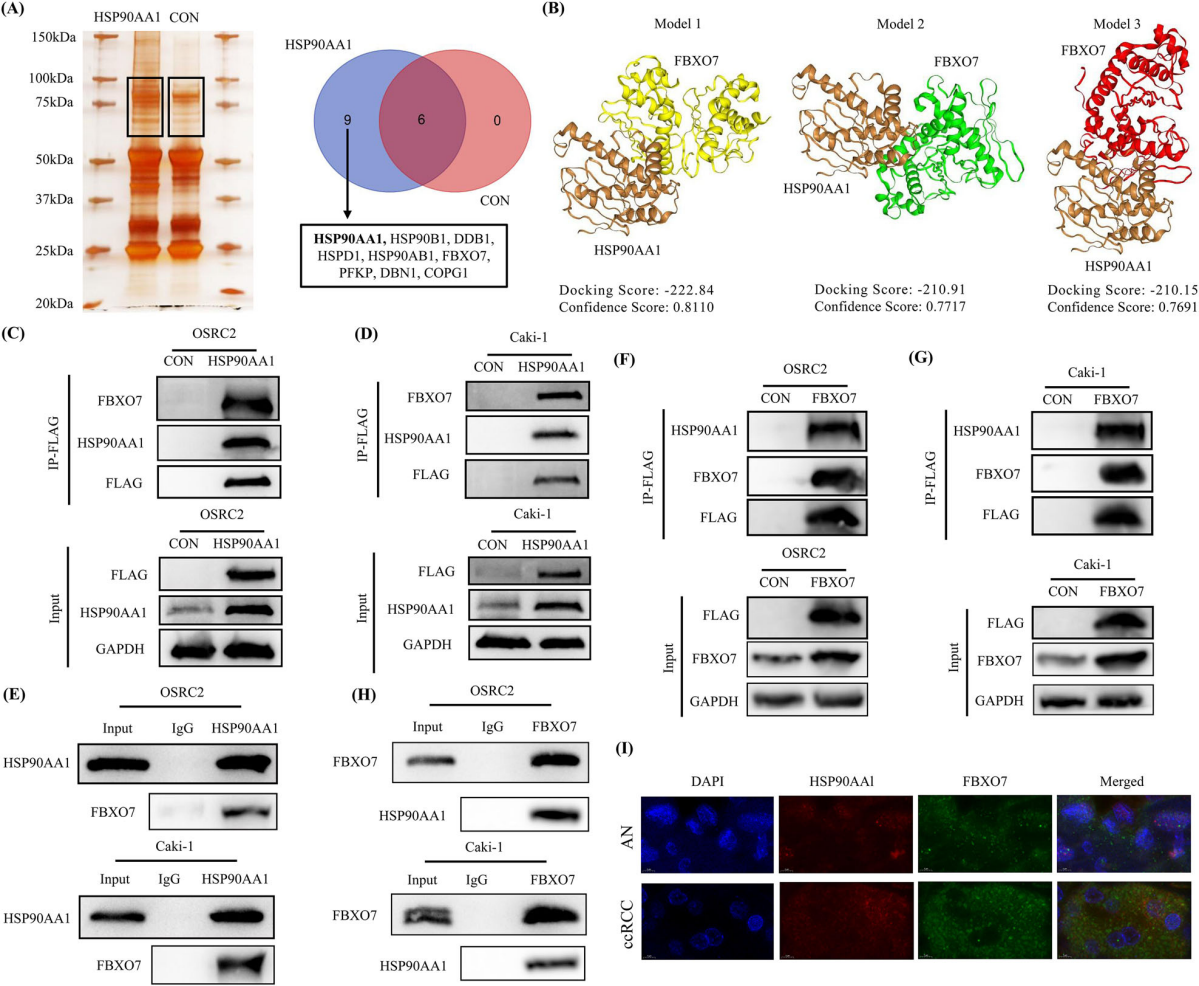
HSP90AA1 bound to the FBXO7 protein. **A** Co-IP, silver staining, and protein mass spectrometry results of HSP90AA1-overexpressing and control ccRCC cells. **B** Prediction of the binding model between the HSP90AA1 protein and the FBXO7 protein via the HDOCK tool. **C**, **D** Exogenous HSP90AA1 IP experiments verified the binding between the HSP90AA1 and FBXO7 proteins in the OSRC2 and Caki-1 cell lines. **E** Endogenous HSP90AA1 IP experiments verified the binding between the HSP90AA1 and FBXO7 proteins. **F**, **G** Exogenous FBXO7 IP experiments verified the binding between the FBXO7 and HSP90AA1 proteins in the OSRC2 and Caki-1 cell lines. **H** Endogenous FBXO7 IP experiments verified the binding between the FBXO7 and HSP90AA1 proteins. **I** Sublocalization detection of HSP90AA1 and FBXO7 protein expression in ccRCC and AN tissues. Scale bar: 5 μm. Repetitions = 3.

To explore the potential mechanism by which HSP90AA1 overexpression inhibits ccRCC proliferation and metastasis, we used Co-IP and silver staining experiments to detect differential protein bands between the HSP90AA1-overexpressing group and the control group. Protein mass spectrometry detection of the differential protein bands revealed 8 potential HSP90AA1-binding proteins (HSP90B1, DDB1, HSPD1, HSP90AB1, FBXO7, PFKP, DBN1, and COPG1) (Fig. 3A). Then, we used the HDOCK molecular docking tool to predict the binding ability between HSP90AA1 and these 8 proteins, and found that the binding ability between HSP90AA1 and the FBXO7 protein was better predictive (Fig. 3B). We subsequently confirmed the binding of the HSP90AA1 protein to the FBXO7 protein in OSRC2 and Caki-1 cells via an exogenous IP experiment (Fig. 3C, D). In addition, endogenous IP experiments also confirmed the binding between the HSP90AA1 and FBXO7 proteins (Fig. 3E). Moreover, we overexpressed the FBXO7 protein in the OSRC2 and Caki-1 cell lines, and both endogenous and exogenous IP experiments confirmed the binding of the FBXO7 protein to the HSP90AA1 protein (Fig. 3F–H). Furthermore, we used immunofluorescence to detect the sublocalization of the HSP90AA1 and FBXO7 proteins in ccRCC and AN tissues. The image acquisition results confirmed that the HSP90AA1 and FBXO7 proteins were expressed mainly in the cytoplasm and that the HSP90AA1 protein and the FBXO7 protein were obviously coexpressed (Fig. 3I).

### FBXO7 expression was decreased in ccRCC and reduced FBXO7 expression predicted poor prognosis

**Fig. 4:**
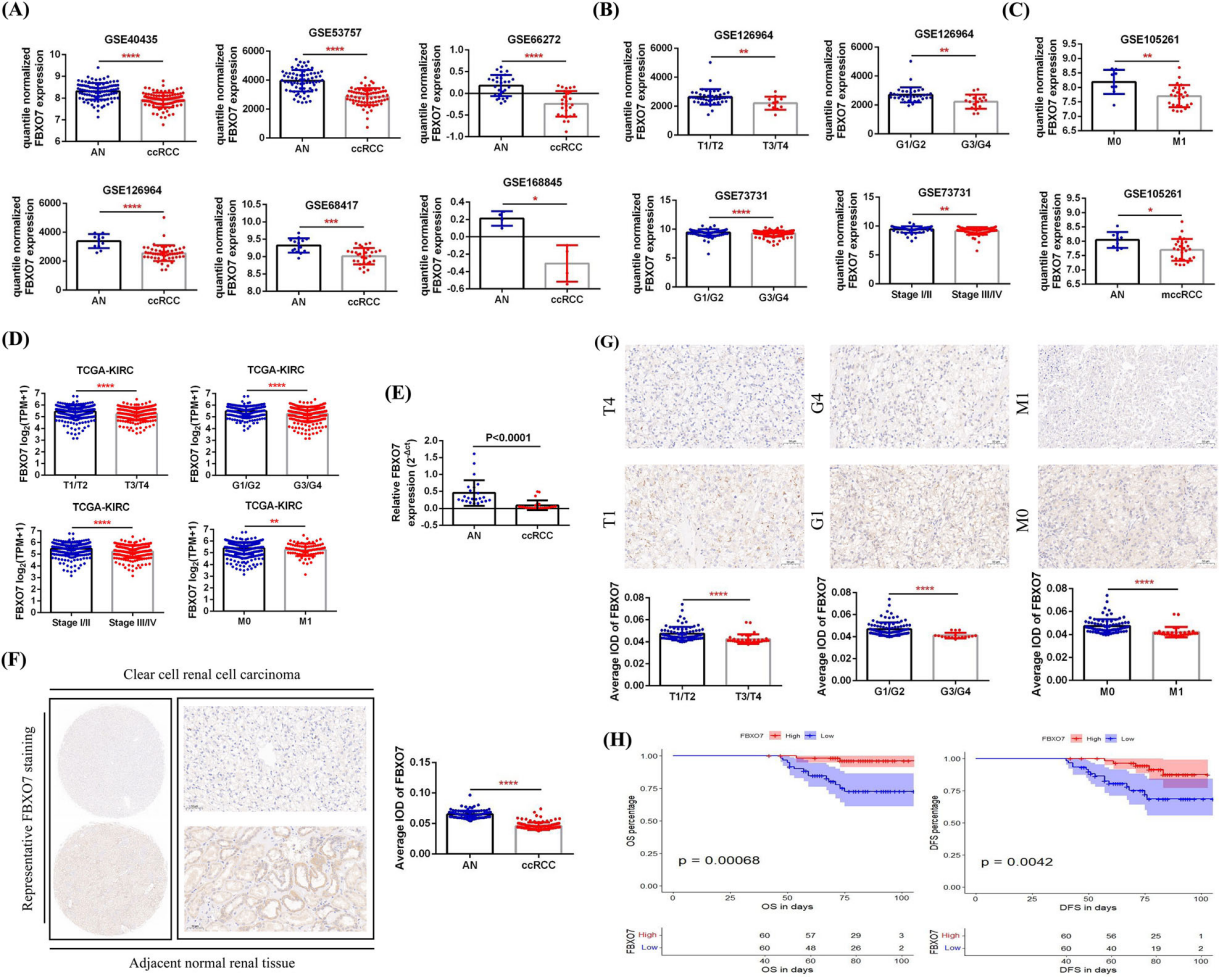
FBXO7 was found to be expressed at low levels in ccRCC in public databases and in clinical samples. **A** Comparison of FBXO7 expression between ccRCC and adjacent normal (AN) tissues in the 6 GSE datasets. **B** Comparison of FBXO7 expression among tumors of different stages and grades in the 2 GSE datasets. **C** Comparison of FBXO7 expression between tumors with and without distant metastasis in the GSE105261 dataset. **D** Comparison of FBXO7 expression in tumors of different stages and grades, as well as between metastatic (M1) and nonmetastatic (M0) tumors, in the TCGA-KIRC database. T1/T2 (*n* = 340, median 5.567), T3/T4 (*n* = 190, median 5.313), G1/G2 (*n* = 241, median 5.575), G3/G4 (*n* = 281, median 5.372), Stage I/II (*n* = 322, median 5.572), Stage III/IV (*n* = 205, median 5.301), M0 (*n* = 440, median 5.509), M1 (*n* = 80, median 5.273). **E** Comparison of the results of qRT-PCR detection of FBXO7 in 24 pairs of ccRCC and AN tissues. **F** Comparison of FBXO7 immunohistochemical staining results for 120 pairs of ccRCC and AN tissues. Scale bar: 50 μm. **G** Comparison of FBXO7 expression in tumors of different stages and grades, as well as between M1 and M0 tumors, on the basis of the immunohistochemistry detection data of these 120 tumors. Scale bar: 50 μm. **H** According to the median expression of FBXO7, these 120 tumors were divided into high- and low-expression groups for prognostic analysis (OS and DFS). ^*^P < 0.05, ^**^P < 0.01, ^***^P < 0.001, ^****^P < 0.0001.

To identify the clinical characteristics of FBXO7 in ccRCC, we compared the mRNA expression of FBXO7 between ccRCC and AN tissues via data from 6 GSE datasets (GSE40435, GSE53757, GSE66272, GSE126964, GSE68417, and GSE168845). Compared with that in AN tissues, FBXO7 expression was significantly lower in ccRCC tissues (Fig. 4A). Analysis of the clinicopathologic features of tumors in 2 GSE datasets (GSE126964 and GSE73731) revealed that decreased FBXO7 expression was associated with increased tumor stage and grade (T3/T4, G3/G4, and Stage III/IV) (Fig. 4B). In addition, decreased FBXO7 expression was closely related to distant tumor metastasis, and its expression in metastatic ccRCCs (M1) was significantly lower than that in nonmetastatic tumors (M0) and their AN tissues on the basis of data from GSE105261 (Fig. 4C). Moreover, we found that lower FBXO7 expression was associated with adverse pathological features of tumors (T3/T4, G3/G4, Stage III/IV, and distant metastasis) on the basis of TCGA-KIRC data (Fig. 4D).

To verify the low expression pattern of FBXO7 in ccRCC, we used RT-qPCR to detect FBXO7 mRNA expression in 24 pairs of ccRCC and AN tissues. The results confirmed that FBXO7 mRNA expression was significantly lower in tumor tissues than in AN tissues (Fig. 4E). Next, we used IHC staining to detect FBXO7 protein expression in 120 pairs of ccRCC and AN tissues. Our results also verified the low expression status of FBXO7 in ccRCC (Fig. 4F). In addition, analysis of the clinicopathologic features of the tumors revealed that lower FBXO7 expression was associated with higher tumor stage and grade (T3/T4 and G3/G4) and distant metastasis (M1) (Fig. 4G). Furthermore, we combined the OS and DFS data of patients for prognostic analysis and determined that the survival time of patients in the high FBXO7 expression group was significantly longer than that of patients in the low FBXO7 expression group (Fig. 4H).

### HSP90AA1 overexpression promoted FBXO7 expression

**Fig. 5:**
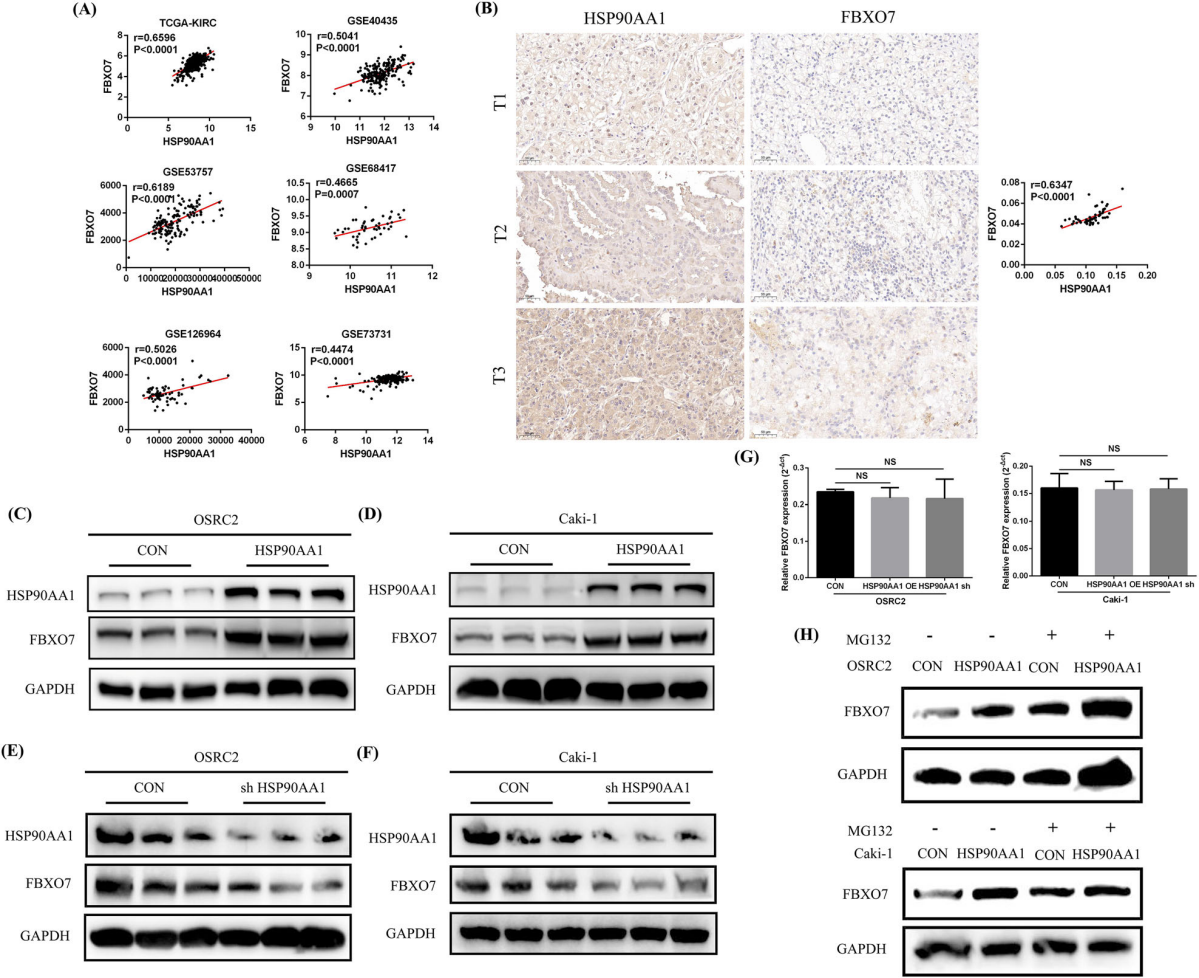
HSP90AA1 overexpression promoted FBXO7 protein expression. **A** The correlation between FBXO7 expression and HSP90AA1 expression was determined on the basis of data from the TCGA-KIRC and 5 GSE datasets. **B** The correlation between HSP90AA1 expression and FBXO7 expression in the paraffin sections of 75 ccRCC tissues. Scale bar: 50 μm. **C**, **D** Comparison of FBXO7 protein expression in the HSP90AA1-overexpressing and control OSRC2 and Caki-1 cell lines. **E**, **F** Comparison of FBXO7 protein expression in HSP90AA1-knockdown and the control OSRC2 and Caki-1 cell lines. **G** Detection of FBXO7 mRNA expression in HSP90AA1-overexpressing and HSP90AA1-knockdown and control OSRC2 and Caki-1 cells by RT-qPCR. **H** The expression of FBXO7 protein in HSP90AA1-overexpressing OSRC2 and Caki-1 cells and their control cells after treatment with MG132. Repetitions = 3.

Given that both HSP90AA1 and FBXO7 are expressed at low levels in ccRCC and have protein-binding abilities with each other, does HSP90AA1 affect the protein expression level of FBXO7? First, we analyzed the correlation between HSP90AA1 expression and FBXO7 expression via data from the TCGA-KIRC and 5 GSE datasets (GSE40435, GSE53757, GSE68417, GSE126964, and GSE73731), and the results revealed a significant positive correlation between HSP90AA1 expression and FBXO7 expression (Fig. 5A). By combining the expression data of HSP90AA1 and FBXO7 in clinical ccRCC paraffin sections, we also confirmed that HSP90AA1 expression was significantly positively correlated with FBXO7 expression (Fig. 5B). In addition, we used Western blot to detect changes in the expression level of FBXO7 in the HSP90AA1-overexpressing and control OSRC2 and Caki-1 cell lines, and the results revealed that FBXO7 expression was significantly upregulated after HSP90AA1 overexpression (Fig. 5C, D). Moreover, after the expression of HSP90AA1 was knocked down, FBXO7 protein expression was also significantly decreased (Fig. 5E, F). However, the RT-qPCR results showed that neither the overexpression nor the knockdown of HSP90AA1 had any significant effect on the expression of FBXO7 mRNA in the OSRC2 and Caki-1 cell lines (Fig. 5G). Then, we treated HSP90AA1-overexpressing OSRC2 and Caki-1 cells, as well as their control cells, with MG132 (a proteasome inhibitor), and examined the expression of FBXO7 protein in them. The results showed that after treatment with MG132, the protein expression of FBXO7 was significantly increased, and the promoting effect of HSP90AA1 on FBXO7 protein expression was significantly inhibited (Fig. 5H).

Thus, the above results suggest that HSP90AA1 may bind to the FBXO7 protein and promote its expression at the post-transcriptional level. The inhibition of HSP90AA1 on the proteasomal pathway-mediated degradation of FBXO7 protein may be an important mechanism for promoting FBXO7 protein expression.

### Knockdown of FBXO7 reversed the inhibition of ccRCC proliferation and metastasis caused by HSP90AA1 overexpression in vitro and in vivo

**Fig. 6:**
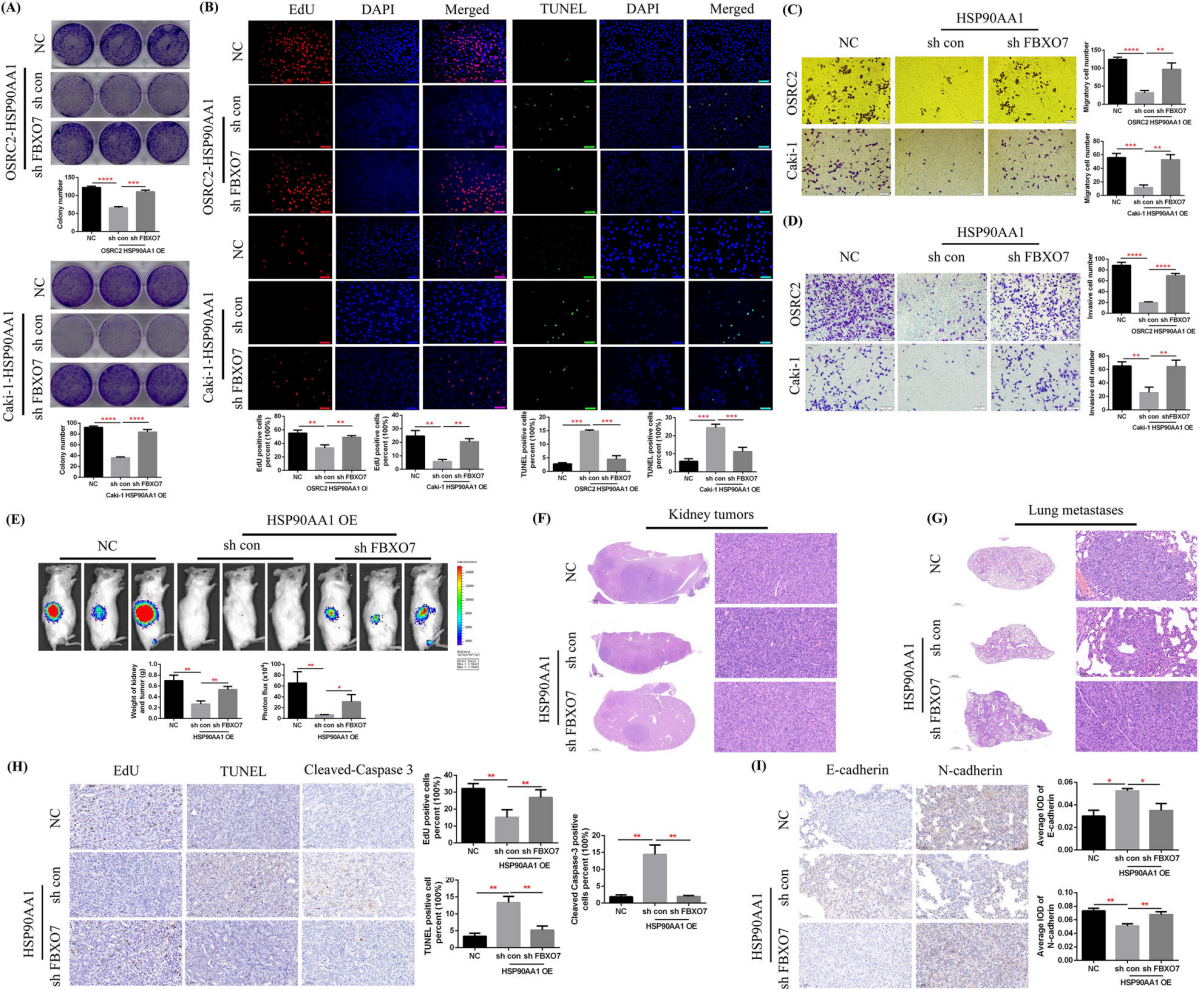
FBXO7 knockdown reversed the inhibition of ccRCC cell proliferation and metastasis caused by HSP90AA1 overexpression. **A** Comparison of the number of colonies formed by FBXO7-knockdown cells and control cells. **B** Comparison of EdU and TUNEL immunofluorescence results between the FBXO7-knockdown group and the control group. **C**, **D** Comparison of the number of migrating and invading FBXO7-knockdown cells and control cells. **E** Comparison of the bioluminescence imaging results between the FBXO7 knockdown group and the control group of mouse renal tumors. **F** H&E staining detection of mouse renal tumors. **G** H&E staining detection of mouse lung metastases. **H** Comparison of EdU, TUNEL, and Cleaved-Caspase 3 expression in the renal tumors of the FBXO7-knockdown group and the control group. **I** Comparison of E-cadherin and N-cadherin expression in the FBXO7-knockdown group and the control group of mouse lung metastases. NC negative control. Scale bar: 50 μm. ^*^P < 0.05, ^**^P < 0.01, ^***^P < 0.001, ^****^P < 0.0001. Repetitions = 3.

To further clarify whether HSP90AA1 affects the proliferation and metastasis ability of ccRCC cells by regulating FBXO7, we knocked down FBXO7 expression on the basis of HSP90AA1 overexpression in the OSRC2 and Caki-1 cell lines. Then, plate colony formation and EdU fluorescence assays were used to detect cell growth and proliferation ability, TUNEL fluorescence assay was used to examine cell apoptosis, and cell migration and invasion experiments were utilized to determine cell invasiveness. Our experimental results confirmed that knocking down FBXO7 expression on the basis of HSP90AA1 overexpression significantly accelerated cell growth (Fig. 6A), enhanced cell proliferation ability, decreased the number of apoptotic cells (Fig. 6B), and promoted cell migration and invasion (Fig. 6C, D).

Similarly, we used a renal orthotopic xenograft tumor growth model to identify the effect of FBXO7 knockdown on ccRCC cell proliferation and metastasis in vivo. We orthotopically injected FBXO7 knockdown or control Caki-1 cells into kidney capsules and monitored tumor growth via weekly bioluminescence imaging. The analysis of bioluminescence imaging data confirmed that the growth rate of renal tumors in the FBXO7-knockdown group was significantly restored (Fig. 6E). H&E staining was then used to detect the pathological structure of the mouse renal tumors and lung metastases (Fig. 6F, G). Subsequently, the EdU and TUNEL methods were used to detect cell proliferation and apoptosis in renal tumors, respectively; IHC staining was used to examine the expression of E-cadherin and N-cadherin in lung metastases. The results revealed that EdU expression was significantly increased, whereas TUNEL and Cleaved-Caspase 3 expression were markedly decreased in the renal tumors of the FBXO7-knockdown group (Fig. 6H). Moreover, E-cadherin expression was significantly reduced, and N-cadherin expression was markedly upregulated in the lung metastases of the FBXO7-knockdown group (Fig. 6I).

In summary, our above results fully demonstrated that HSP90AA1 affects the proliferation and metastasis ability of ccRCC cells by regulating the expression of FBXO7.

### HSP90AA1 overexpression upregulated CADM1 expression and inhibited the PI3K-AKT pathway by regulating FBXO7

**Fig. 7:**
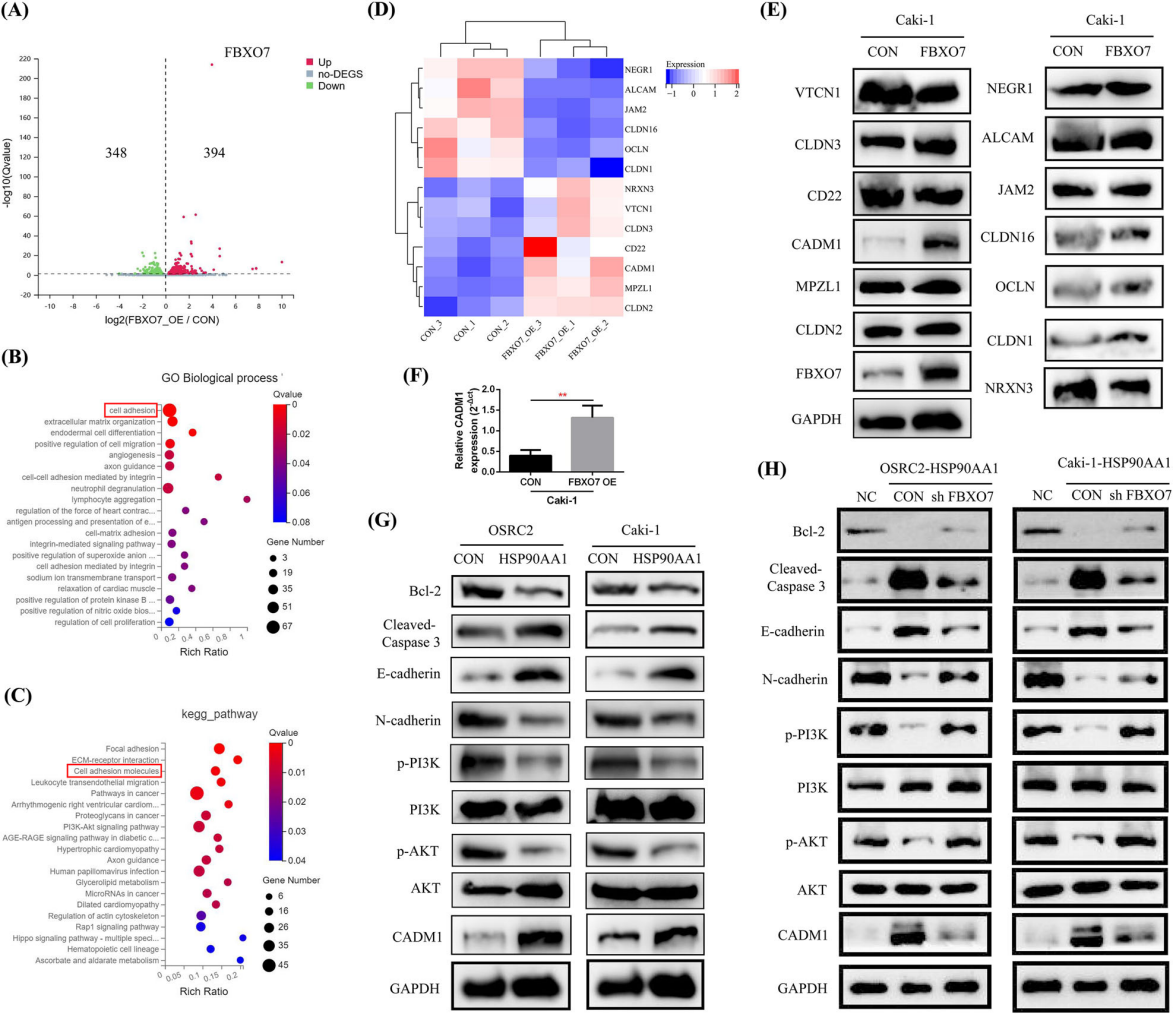
HSP90AA1 promoted CADM1 expression and suppressed the PI3K-AKT signaling pathway by regulating FBXO7. **A** Differential gene volcano plot of FBXO7-overexpressing and control Caki-1 cells detected via RNA-seq. **B**, **C** GO biological process and KEGG pathway enrichment analysis of the DEGs. **D** Differential gene expression heatmap of the cell adhesion molecules KEGG pathway. **E** Expression detection of these 13 differential molecules (CLDN2, MPZL1, CADM1, CD22, CLDN3, VTCN1, NRXN3, CLDN1, OCLN, CLDN16, JAM2, ALCAM, and NEGR1) in FBXO7-overexpressing and control Caki-1 cells by western blot. **F** Detection of CADM1 mRNA expression in FBXO7-overexpressing and control Caki-1 cells by RT-qPCR. **G** Detection of CADM1, p-AKT, p-PI3K, N-cadherin, E-cadherin, Cleaved-Caspase 3, and Bcl-2 expression in HSP90AA1-overexpressing and control OSRC2 and Caki-1 cells via western blot. **H** Detection of CADM1, p-AKT, p-PI3K, N-cadherin, E-cadherin, Cleaved-Caspase 3, and Bcl-2 expression in FBXO7-knockdown cells and control OSRC2 and Caki-1 cells via western blot. NC negative control. Repetitions = 3.

To explore the downstream molecular regulatory mechanism of FBXO7, we performed RNA-seq on FBXO7-overexpressing and control Caki-1 cells and analyzed the DEGs (Differentially Expressed Genes). Compared with those in the control group, 394 significantly upregulated genes and 348 significantly downregulated genes were found in the FBXO7-overexpressing group (Fig. 7A). GO biological process and KEGG pathway enrichment analyses of the DEGs revealed a close correlation between FBXO7 expression and cell adhesion (Fig. 7B, C). The gene expression heatmap revealed that among these 13 DEGs in the cell adhesion molecules pathway, 7 genes (CLDN2, MPZL1, CADM1, CD22, CLDN3, VTCN1, and NRXN3) were upregulated, and 6 genes (CLDN1, OCLN, CLDN16, JAM2, ALCAM, and NEGR1) were downregulated after FBXO7 overexpression (Fig. 7D). We subsequently validated the protein expression of these 13 molecules via Western blot and found that only the CADM1 protein was significantly upregulated after FBXO7 overexpression, whereas no significant changes in the expression of the other molecules were detected (Fig. 7E). Moreover, the RT-qPCR detection result also confirmed that overexpression of FBXO7 significantly increased the mRNA expression of CADM1 in Caki-1 cells (Fig. 7F).

Cell adhesion molecule 1 (CADM1) is a member of the immunoglobulin superfamily, and increased CADM1 expression can inhibit tumor progression by suppressing the PI3K-AKT signaling pathway [13]. Next, we detected the expression of CADM1, p-PI3K, p-AKT, N-cadherin, E-cadherin, Cleaved-Caspase 3, and Bcl-2 in HSP90AA1-overexpressing and the control OSRC2 and Caki-1 cell lines. The results revealed that after HSP90AA1 overexpression, the expression of CADM1, E-cadherin, and Cleaved-Caspase 3 was significantly increased, whereas the expression of p-PI3K, p-AKT, N-cadherin, and Bcl-2 was markedly decreased (Fig. 7G). Moreover, we detected changes in the expression of these molecules after knocking down FBXO7 expression in the context of HSP90AA1 overexpression, and the results confirmed that the decreased expression of p-PI3K, p-AKT, N-cadherin, and Bcl-2 was significantly restored, whereas the increased expression of CADM1, E-cadherin, and Cleaved-Caspase 3 was significantly reduced (Fig. 7H).

Therefore, our above results demonstrated that HSP90AA1 may promoted CADM1 expression and inhibited PI3K-AKT pathway by upregulating FBXO7.

## Discussion

In the past few decades of research, a series of functions of HSP90 as a highly conserved molecular chaperone have been identified, which can contribute to the correct folding of proteins, maintain their stability under cellular stress, and aid in the degradation of damaged or misfolded proteins [14, 15]. Many of these proteins are involved in important physiological processes such as cell proliferation, differentiation, and survival [16]. In cancer cells, the expression of HSP90 is usually elevated, and its functional dependence is significantly enhanced due to the increased burden of mutant or overexpressed cancer proteins [17]. Based on this characteristic, HSP90 inhibitors have been widely developed and clinically evaluated. However, HSP90AA1 as an inducible isoform of HSP90, there is currently almost no research on HSP90AA1 in RCC.

In this study, we fully confirmed the low expression of HSP90AA1 in ccRCC through analysis of public databases and validation via IHC staining of clinical ccRCC samples. In addition, low expression of HSP90AA1 was associated with tumor metastasis. Moreover, cellular functional experiments and mouse animal models revealed that HSP90AA1 overexpression significantly restrained the proliferation and metastasis ability of ccRCC cells both in vitro and in vivo. Therefore, our above results revealed for the first time that HSP90AA1 acts as a tumor suppressor in ccRCC.

Moreover, given that HSP90 is capable of regulating the activation processes of various proteins within the cell, our study also revealed that HSP90AA1 can bind to the FBXO7 protein and promote its protein expression, but did not affect its RNA expression level. Sublocalization staining results confirmed that these two proteins were coexpressed in the cytoplasm of ccRCC cells. Moreover, after treatment with MG132, the expression of FBXO7 protein was significantly increased, while the promoting effect of HSP90AA1 on FBXO7 protein expression was significantly inhibited. This indicates that HSP90AA1 is highly likely to inhibit the proteasomal pathway degradation of FBXO7 protein. Similar to our research findings, a previous study has indicated that HSP90AA1 interacts with NME1 and increases NME1 lifetime by impeding its ubiquitin-proteasome-mediated degradation and suppresses breast cancer metastasis [12]. However, the specific regulatory mechanisms by which HSP90AA1 promotes the expression of the FBXO7 protein remain to be further elucidated. The exact mechanism of this process still requires further investigation.

F-box proteins can affect multiple cellular processes, including tumor growth, proliferation, invasion, and metastasis, by regulating the cell cycle, DNA damage repair, and epithelial-mesenchymal transition (EMT) [18–22]. Emerging evidence suggests that FBXO7, an F-box protein, plays important regulatory roles in various tumors and could serve as a target for tumor therapy. For example, FBXO7 is significantly downregulated in HCC tissues, and the downregulation of PRMT1 weakens the methylation and activation of arginine in PHGDH, leading to impaired serine synthesis, accumulation of reactive oxygen species, and inhibition of HCC cell growth [23]; FBXO7 acts as a tumor suppressor in endometrial cancer by inhibiting INF2-related mitochondrial fission [24]; FBXO7 promotes mesenchymal properties and chemotherapy resistance in glioblastoma by controlling Rbfox2-mediated alternative splicing, and the loss of FBXO7 enhances the sensitivity of mouse tumor xenografts to chemotherapy [25]. Thus, it can be seen that FBXO7 did not show the same inhibitory or promoting effect in different tumors. However, there is currently limited research on FBXO7 in RCC, and its expression characteristics, biological functions, and mechanisms of action remain unclear.

In the present study, we confirmed the low expression of FBXO7 in ccRCC through analysis of public databases and validation via RT-qPCR and IHC staining of clinical ccRCC samples. Moreover, low FBXO7 expression was also associated with adverse pathological characteristics of tumors, and patients in the high FBXO7 expression group had a better prognosis than those in the low FBXO7 expression group. In addition, after FBXO7 expression was knocked down in the context of HSP90AA1 overexpression, the phenotypes inhibited caused by HSP90AA1 overexpression were significantly restored in vitro and in vivo. Thus, our results suggested that HSP90AA1 acts as a tumor suppressor in ccRCC by regulating FBXO7. Consistent with our findings, previous studies have shown that FBXO7 functions as a tumor suppressor in various tumors, including HCC [23] and endometrial carcinoma [24].

Currently, most studies suggest that FBXO7 primarily functions in cancers by ubiquitinating its substrate proteins and subsequently degrading them through the proteasome pathway [26, 27]. However, in this study, we used RNA-seq to screen the DEGs between the FBXO7-overexpressing group and the control group and performed GO and KEGG pathway enrichment analyses. After Western blot validation, we found that FBXO7 overexpression significantly upregulated the mRNA and protein expression of the key molecule CADM1 in the cell adhesion molecules KEGG pathway. The cell adhesion molecule CADM1 is involved in cell adhesion and signal transduction and has a regulatory effect on the occurrence and development of tumors. Its upregulation can promote tumor cell apoptosis and inhibit malignant proliferation. In addition, CADM1 participates in regulating signaling pathways such as EMT and the PI3K-AKT pathway, thus playing an important role in inhibiting invasion and migration [13, 28, 29].

In this study, we found that HSP90AA1 overexpression significantly increased CADM1 expression, while the expression of p-PI3K, p-AKT, N-cadherin, and Bcl-2 was significantly reduced, and the expression of Cleaved-Caspase 3 and E-cadherin was markedly increased. In addition, after FBXO7 expression was knocked down in the context of HSP90AA1 overexpression, the expression of p-PI3K, p-AKT, N-cadherin, and Bcl-2 was significantly upregulated, whereas the expression of CADM1, Cleaved-Caspase 3, and E-cadherin was inhibited. Thus, our findings confirmed that HSP90AA1 may inhibit ccRCC proliferation and metastasis by affecting CADM1 expression and the PI3K-AKT signaling pathway through the regulation of FBXO7.

However, since FBXO7 is a member of the F-box protein family and is a component of the SCF (SKP1-CUL1-F-box protein) E3 ubiquitin-protein ligase complex, it is usually capable of recognizing specific substrate proteins and promoting their ubiquitination, thereby regulating the degradation or function of these proteins [30]. Therefore, whether FBXO7 regulates the protein stability of certain transcription factors and thereby exerts its tumor-suppressing effect in RCC deserves further research exploration in the near future. Furthermore, the biological functions of FBXO7 in RCC, as well as the downstream regulatory mechanism of FBXO7 on CADM1 expression, still require further research and exploration. This includes verifying whether CADM1 can mediate the downstream effects of FBXO7 and HSP90AA1 on the PI3K-AKT signaling pathway and cell phenotype by knocking down CADM1.

Interestingly, a recent study has shown that Mulberrin (a natural flavonoid featured with two isopentenyl groups) can inhibit the PI3K-AKT pathway and the EMT process by reducing HSP90AA1 expression, thereby suppressing the proliferation, migration, and invasion abilities of gastric cancer cells (GC) [31]. This seems contradictory to our finding that HSP90AA1 inhibits the PI3K-AKT pathway in ccRCC. However, this study indicated that HSP90AA1 was significantly overexpressed in GC, while we have fully confirmed that the expression level of HSP90AA1 in ccRCC was significantly lower compared to that in the AN tissues. From this, it can be seen that the basic expression of HSP90AA1 varies among different tumors, and its functions and regulatory mechanisms may also differ in various tumors. Currently, HSP90 is widely regarded as a promising target for the treatment of various cancers [32]. Preclinical data also indicate that HSP90 inhibitors have certain inhibitory effects on certain tumors [33]. However, due to the limited efficacy, more evidence is still needed to prove its effectiveness in clinical practice. However, in order to explore the potential application of HSP90 inhibitors in RCC, we first need to clarify the expression characteristics and regulatory mechanisms of other members of the HSP90 family in RCC, and determine whether they play a role in tumor suppression or promotion. At the same time, a large number of animal experiments are needed to provide more preclinical data of HSP90 inhibitors in RCC. It is hoped that these research projects can be completed in the near future.

**Fig. 8:**
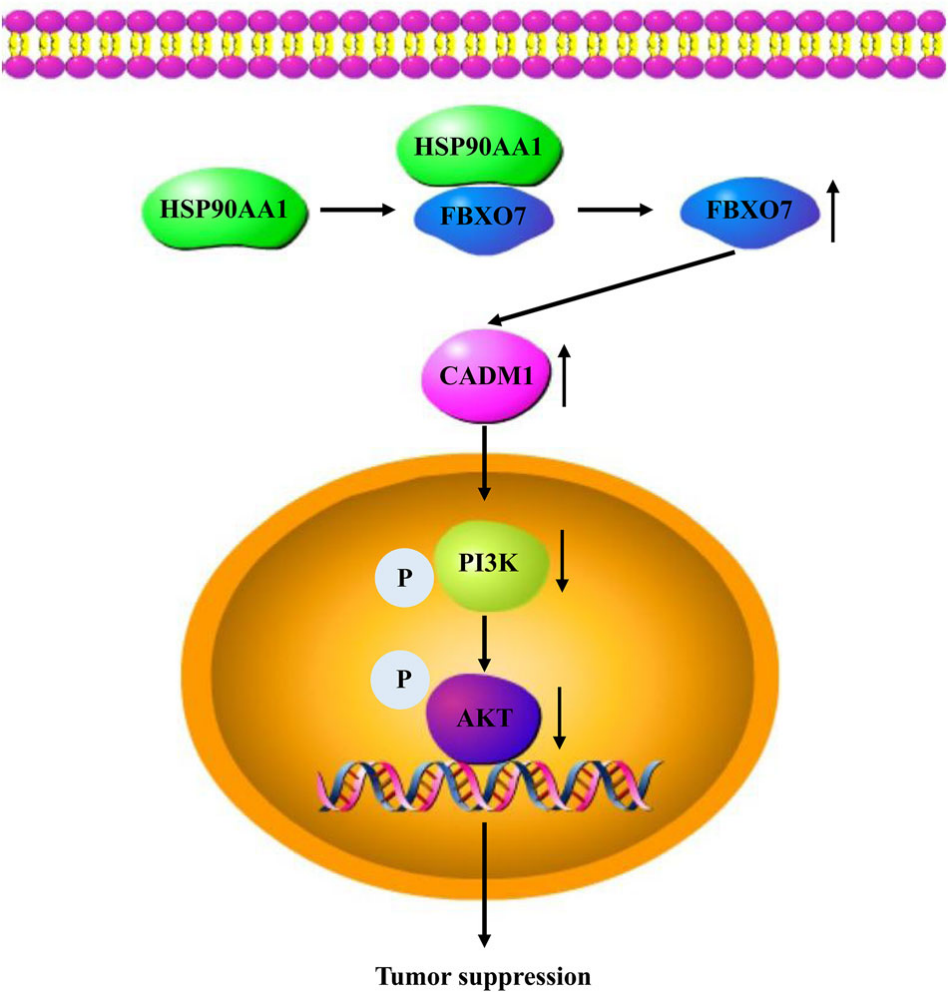
The mechanism by which HSP90AA1 inhibits the proliferation and metastasis of ccRCC by interacting with FBXO7 to promote CADM1 expression and suppress the PI3K-AKT signaling pathway.

In conclusion, our study revealed the low expression pattern of HSP90AA1 in ccRCC, validated its clinical prognostic value, and revealed a new mechanism by which HSP90AA1 inhibits the proliferation and metastasis of ccRCC by interacting with FBXO7 to promote CADM1 expression and suppress the PI3K-AKT signaling pathway (Fig. 8).

## Materials and methods

### Public database mining

Eight GSE datasets (GSE40435, GSE53757, GSE66272, GSE126964, GSE68417, GSE168845, GSE73731, and GSE105261) containing ccRCC tissue transcriptome analysis data were obtained from the GEO (Gene Expression Omnibus) database (https://www.ncbi.nlm.nih.gov/geo). Transcriptome sequencing data from ccRCC tissues and survival time information from patients in the TCGA-KIRC (The Cancer Genome Atlas-Kidney Renal Clear Cell Carcinoma) database were obtained from https://www.cancer.gov/ccg/research/genome-sequencing/tcga.

### Clinical sample collection

Twenty-four pairs of fresh ccRCC and adjacent normal (AN) tissues and paraffin-embedded tissue sections of 120 pairs of ccRCC and AN tissues were obtained from the Department of Urology, The First Affiliated Hospital, Zhejiang University School of Medicine. In addition, we obtained survival time information for these 120 ccRCC patients through follow-up. The study was approved by the IIT ethics review committee of the hospital (ethical approval number: 2024 Research No. 0921), and we obtained all the informed consent forms signed by patients or their families.

### Cell culture and transfection

Two control cell lines (HK2 and HEK293) and 5 ccRCC lines (786-O, A498, RCC4, OSRC2, and Caki-1) were purchased from the ATCC (American Type Culture Collection) cell bank. All the cells were cultured in DMEM containing 10% fetal bovine serum. HSP90AA1 overexpression and knockdown and FBXO7 overexpression and knockdown viruses were packaged via a three-plasmid system (target plasmid, pMD2. G, and psPAX2) in 293T cells. The HSP90AA1 and FBXO7 overexpression plasmids contained the expression sequence of the tag protein FLAG. The targeting sequences of HSP90AA1 and FBXO7 knockdown plasmids were as follows: HSP90AA1 sh1 TATGGCATGACAACTACTTTA, HSP90AA1 sh2 TACTTGGAGGAACGAAGAATA, FBXO7 sh1 GCCACATTCATTAGAGACCTT, FBXO7 sh2 GCTGACTGTTCTGATGCCAAT.

### Immunohistochemical (IHC) staining

After the paraffin sections were dewaxed in water, hydrogen peroxide was used to block endogenous peroxidase activity and to perform antigen repair. Then, the sections were incubated with primary antibodies overnight at 4 °C. The specific primary antibodies used were as follows: anti-HSP90AA1 (Sigma, SAB4501461, 1:400), anti-FBXO7 (Abcam, ab167278, 1:100), anti-E-cadherin (Abcam, ab40772, 1:500), anti-N-cadherin (Abcam, ab19348, 1:1000), and anti-Cleaved-Caspase 3 (CST, #9661, 1:400). Image-Pro Plus (IPP) software was used to determine the average integrated optical density (IOD) values of HSP90AA1, FBXO7, E-cadherin, and N-cadherin staining, as well as the percentages of TUNEL-, EdU-, and Cleaved-Caspase 3-positive cells.

### Western blot

After SDS‒PAGE electrophoresis, membrane transfer (PVDF membrane), and blocking, the membranes were incubated with primary antibody at 4 °C. The specific primary antibodies used were as follows: anti-HSP90AA1 (Sigma, SAB4501461, 1:1000), anti-FBXO7 (Abcam, ab167278, 1:1000), anti-CLDN2 (Proteintech, 26912-1-AP, 1:2000), anti-MPZL1 (Proteintech, 29784-1-AP, 1:2000), anti-CADM1 (Proteintech, 14335-1-AP, 1:1000), anti-CD22 (Proteintech, 66103-1-Ig, 1:3000), anti-CLDN3 (ABclonal, A11650, 1:1000), anti-VTCN1 (Proteintech, 12080-1-AP, 1:1000), anti-NRXN3 (Proteintech, 21849-1-AP, 1:1000), anti-CLDN1 (Proteintech, 13050-1-AP, 1:2000), anti-OCLN (Proteintech, 66378-1-Ig, 1:10000), anti-CLDN16 (Proteintech, 27638-1-AP, 1:1000), anti-JAM2 (Proteintech, 12972-1-AP, 1:1000), anti-ALCAM (Proteintech, 21972-1-AP, 1:5000), anti-NEGR1 (Proteintech, 13674-1-AP, 1:1000), anti-AKT (Proteintech, 10176-2-AP, 1:5000), anti-p-AKT (Proteintech, 66444-1-Ig, 1:4000), anti-PI3K (Proteintech, 60225-1-Ig, 1:10000), anti-p-PI3K (Abcam, ab278545, 1:1000), anti-N-cadherin (1:5000, Abcam, ab76011), anti-E-cadherin (Proteintech, 13674-1-AP, 1:1000), anti-Cleaved-Caspase 3 (CST, #9661,1:1000), anti-Bcl-2 (1:2000, 12789-1-AP, Proteintech), and anti-GAPDH (Proteintech, 60004-1-Ig, 1:10000). Then, the PVDF membrane was subjected to enhanced chemiluminescence (ECL) imaging after being washed three times with TBS-T washing solution, and the raw data were also saved. The experiments were conducted with three biological replicates.

### Cell growth, proliferation, and apoptosis detection

A plate colony formation assay was used to detect the relative growth ability of ccRCC cells. EdU fluorescence and DAB staining methods were used to detect proliferating cells in cell slides and paraffin sections, respectively. TUNEL fluorescence and DAB staining methods were used to detect apoptotic cells in cell slides and paraffin sections, respectively. All procedures were performed according to the instructions of the test kits (Beyotime, C0081S, C0085S, C1086, and C1091). The experiments were conducted with three biological replicates.

### Cell migration and invasion experiments

The cell migration and invasion experiments were mainly used to detect the invasiveness of ccRCC cells in vitro, and the specific experimental steps have been described in detail in previously published articles [34]. The experiments were conducted with three biological replicates.

### Co-IP (co-immunoprecipitation) experiments

First, the total protein of the HSP90AA1-overexpressing and control cell lines was extracted separately and quantified via the BCA method, and 100 µl of protein supernatant was used as the input control. For exogenous IP, equal volumes and masses of protein supernatant from each group were incubated with 50 µl of anti-FLAG magnetic beads (Sigma) overnight at 4 °C. The next day, the protein mixture was discarded, the magnetic beads were washed three times with protein mixture, 2× protein loading buffer was added, the mixture was boiled for 5 min, and the protein loading buffer was collected. Subsequently, Western blot was used to detect the protein expression of FLAG and HSP90AA1 or FBXO7 in the IP samples. After the expression of the FLAG and HSP90AA1 or FBXO7 proteins was successfully verified, silver staining was used to detect differential protein bands between the HSP90AA1 protein overexpression group and the control group, followed by protein mass spectrometry detection.

For endogenous IP, an equal volume of protein supernatant from the same sample was incubated with anti-HSP90AA1 or anti-FXO7 protein antibody overnight at 4 °C. The next day, 50 µl of protein A/G magnetic beads (Sigma) were added to the antigen‒antibody complex. After the mixture was incubated at room temperature for 4 h, the protein mixture was discarded, the magnetic beads were washed three times with protein lysate, 2× protein loading buffer was added, the mixture was boiled for 5 min, and the protein loading buffer was collected for western blot analysis. The experiments were conducted with three biological replicates.

### Molecular docking model prediction

The HDOCK online tool (http://hdock.phys.hust.edu.cn/) was used to construct a protein binding model between the HSP90AA1 and FBXO7 proteins and predict binding sites. Docking score: A more negative docking score indicates a more likely binding model. Confidence score: When the confidence score is greater than 0.7, two molecules are likely to combine.

### RT-qPCR

First, the total RNA of 24 pairs of ccRCC and AN tissues and HSP90AA1-overexpressing and FBXO7-overexpressing cell lines was extracted via Trizol reagent, followed by reverse transcription to obtain cDNA, and finally RT-qPCR detection was performed via the One Step RT-qPCR SYBR Green Kit. The upstream and downstream primer sequences were as follows: FBXO7 forward primer GATTCAGAGCATTCTTCACTCCA, FBXO7 reverse primer GCCCTAACATACTGTCGTCATTC; CADM1 forward primer ATGGCGAGTGTAGTGCTGC, CADM1 reverse primer GATCACTGTCACGTCTTTCGT. The experiments were conducted with three biological replicates.

### Immunofluorescence detection

After the paraffin sections were dewaxed in water, hydrogen peroxide was used to block endogenous peroxidase activity and to perform antigen repair, and the sections were incubated with primary antibodies overnight at 4 °C. The specific primary antibodies used were as follows: anti-HSP90AA1 (Sigma, SAB4501461, 1:100) and anti-FBXO7 (Abcam, ab167278, 1:100). After the sections were washed three times, they were incubated with a mouse secondary antibody with green fluorescence and a rabbit secondary antibody with red fluorescence at room temperature for 2 h. After counterstaining with DAPI staining solution, image acquisition was performed via confocal laser microscopy to clarify the subcellular localization of the proteins.

### RNA sequencing (RNA-seq)

RNA-seq was performed by Beijing Liuhe Huada Gene Technology Co., Ltd. The DESeq2 method was used to analyze the DEGs (Differentially Expressed Genes), and the screening criteria for DEGs were Qvalue (Adjusted *P* value) ≤ 0.05. GO (Gene Ontology) and KEGG (Kyoto Encyclopedia of Genes and Genomes) pathway enrichment analyses were also performed, the basic function “phyper” in R language was used to calculate the *P*-value, then multiple test correction was performed on these *P*-value, and finally the screening threshold was set as the *Q*-value ≤ 0.05.

### Animal experiments

Thirty 6-week-old severely immunodeficient B-NDG mice were randomly divided into five groups (*n* = 6). The HSP90AA1-overexpressing group and control group cells, as well as the FBXO7-knockdown group and control group cells, were subsequently orthotopically injected into the kidney capsules to construct a mouse renal orthotopic xenograft tumor growth model. The growth of mouse renal tumors was monitored through weekly bioluminescence imaging, and the specific method has been described in a previously published article [35]. Three hours before each mouse was euthanized, an EdU drug solution (50 mg/kg) was injected intraperitoneally for subsequent detection of EdU expression in paraffin sections. This animal experiment was approved by the Experimental Animal Ethics Committee of the First Affiliated Hospital of Zhejiang University School of Medicine (Ethical approval number: 2024--1278). No blinding performed for the animal studies.

### Statistical analyses

The differences in HSP90AA1 and FBXO7 expression between two groups were examined via nonparametric Mann‒Whitney test or Student’s *t* test. Kaplan‒Meier survival curves and log-rank tests were used to compare the prognostic differences between ccRCC patients in the high- and low-HSP90AA1 and FBXO7 expression groups. The correlation between HSP90AA1 expression and FBXO7 expression was examined via the Pearson method. All the statistical tests were two-tailed, and a *P* value < 0.05 was considered statistically significant. All of these data statistical analyses were completed using GraphPad Prism 6 and R language.

## Supplementary information

Original Western blot picture

## Data Availability

The data analyzed and generated in this study are all included in this manuscript.
