# Supplementary material for: HSP90AA1 restrains clear cell renal cell carcinoma progression by promoting CADM1 expression and suppressing the PI3K-AKT pathway through interaction with FBXO7
Source: Cell Death Discov. 2026 Jan 8;12:6. doi: 10.1038/s41420-025-02848-4 (PMC12783301; doi:10.1038/s41420-025-02848-4)
Supplement: Supplementary file 1 — Original Western blot picture [file 41420_2025_2848_MOESM1_ESM.pdf]

**The full uncropped Blots images**

Fig. 1E

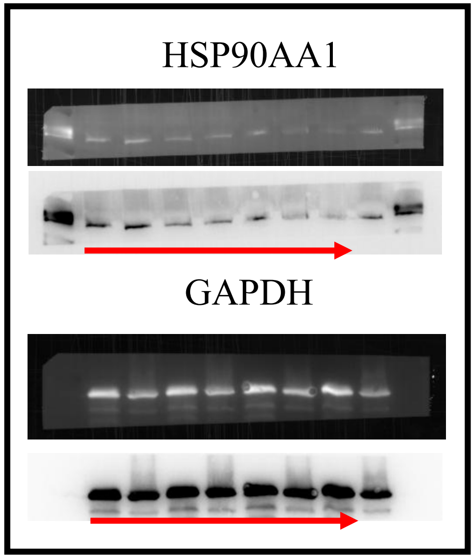


Fig. 1F

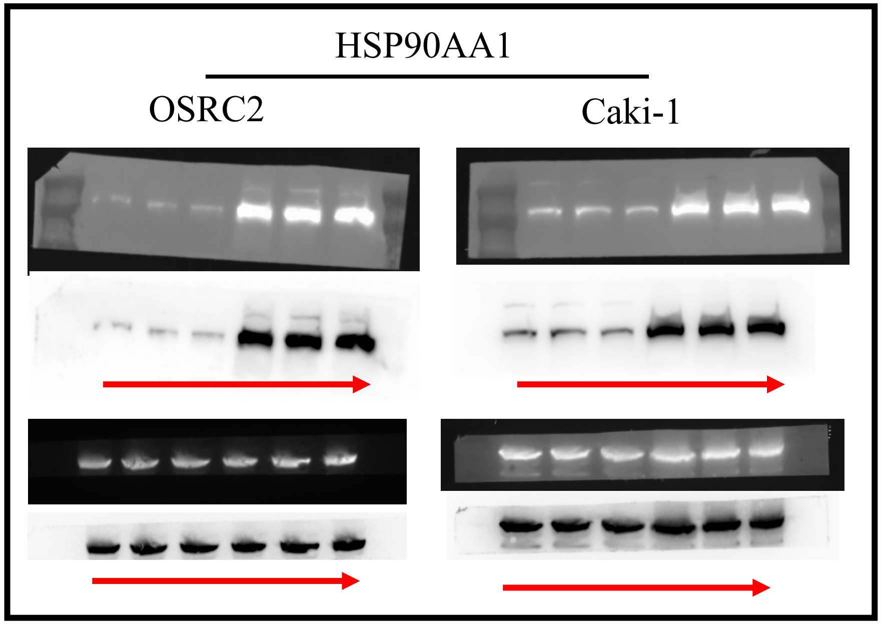

Fig. 3C

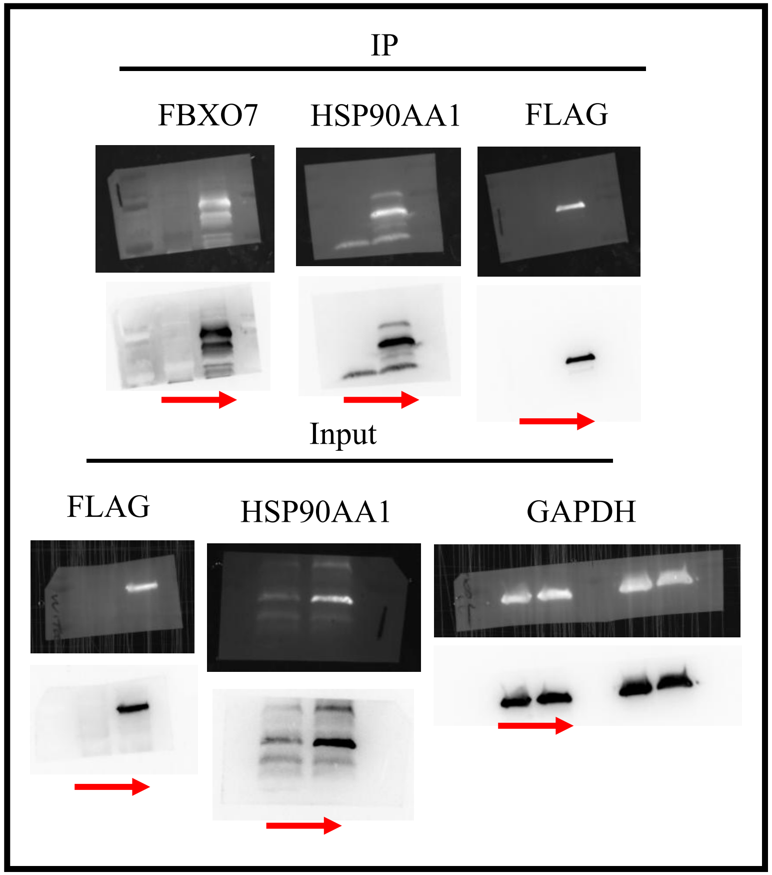
IP
FBXO7
HSP90AA1
FLAG
Input
FLAG
HSP90AA1
GAPDH

Fig. 3D

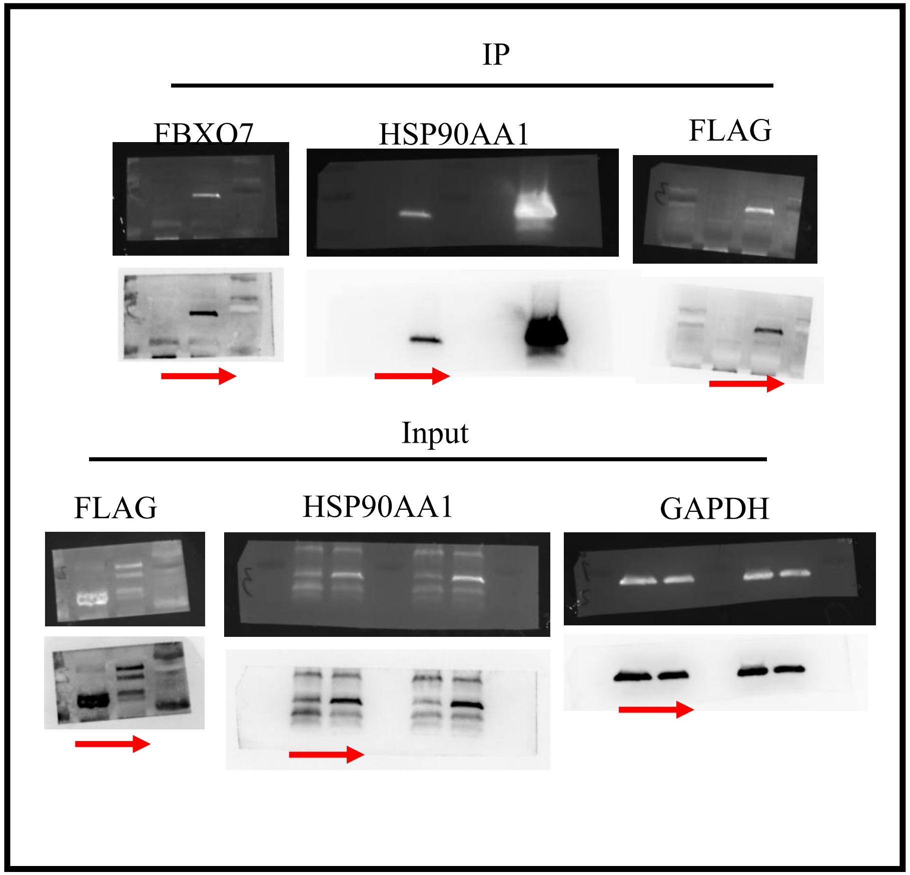
IP
FBXO7
HSP90AA1
FLAG
Input
FLAG
HSP90AA1
GAPDH

Fig. 3E

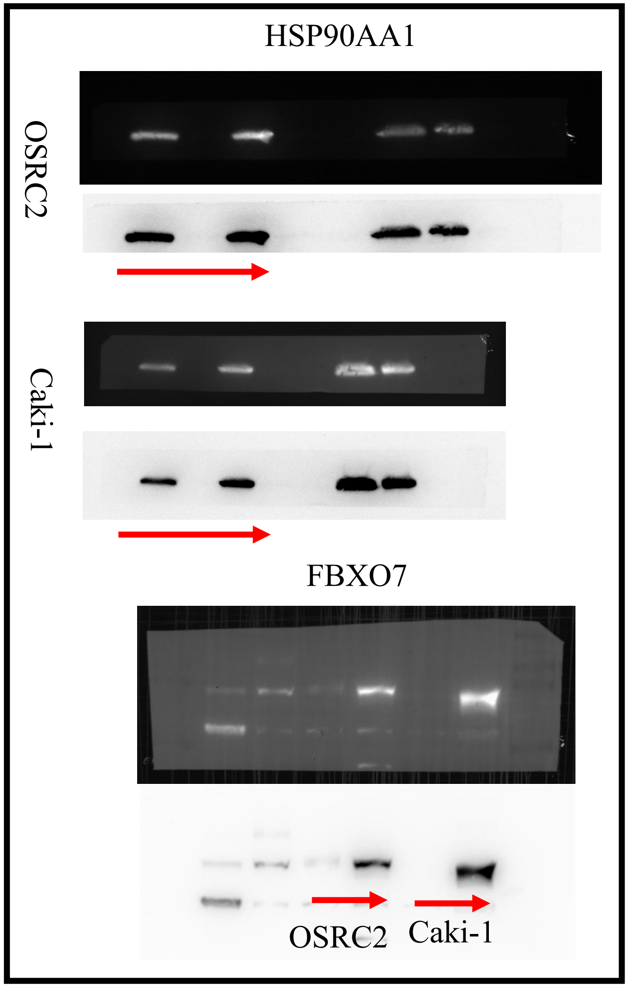
HSP90AA1
OSRC2
Caki-1
FBXO7
OSRC2
Caki-1

Fig. 3F

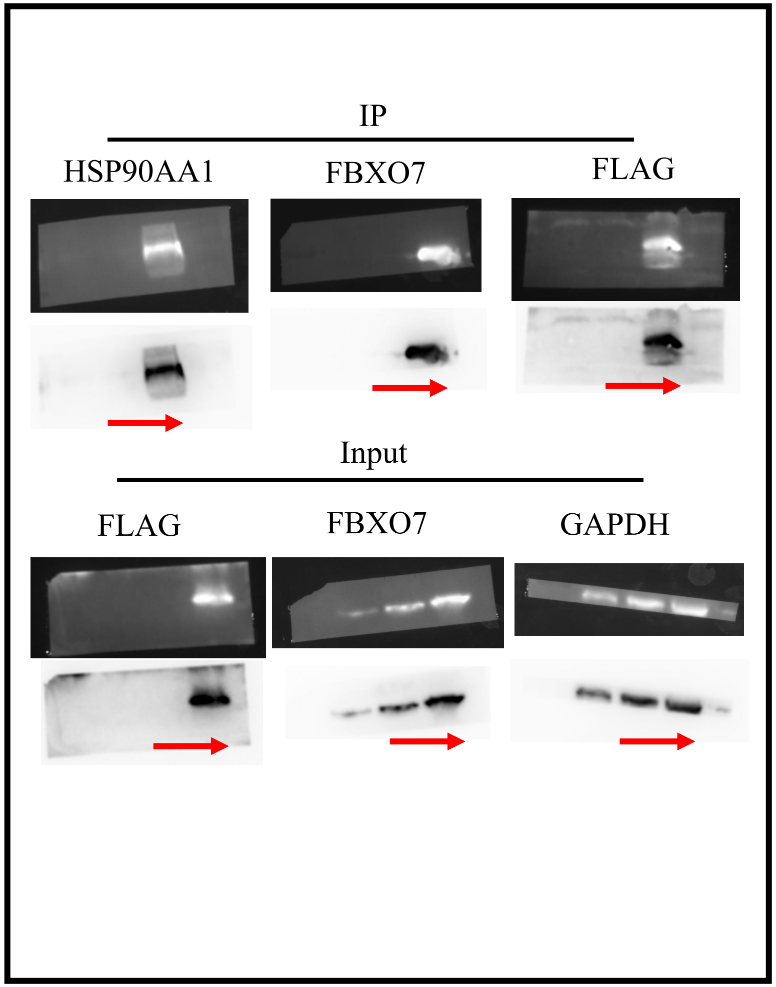
IP
HSP90AA1
FBXO7
FLAG
Input
FLAG
FBXO7
GAPDH

Fig. 3G

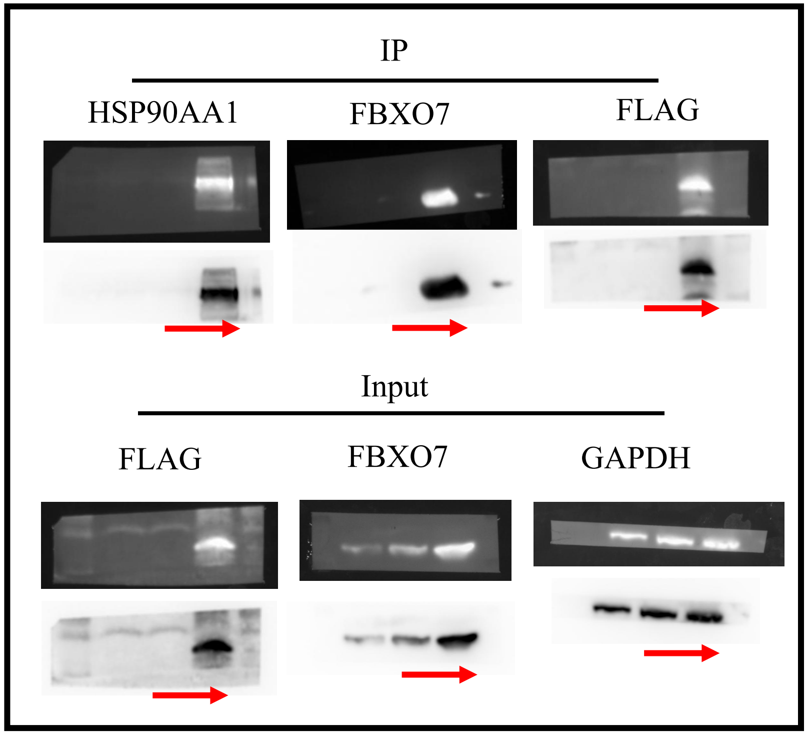
IP
HSP90AA1
FBXO7
FLAG
Input
FLAG
FBXO7
GAPDH

Fig. 3H

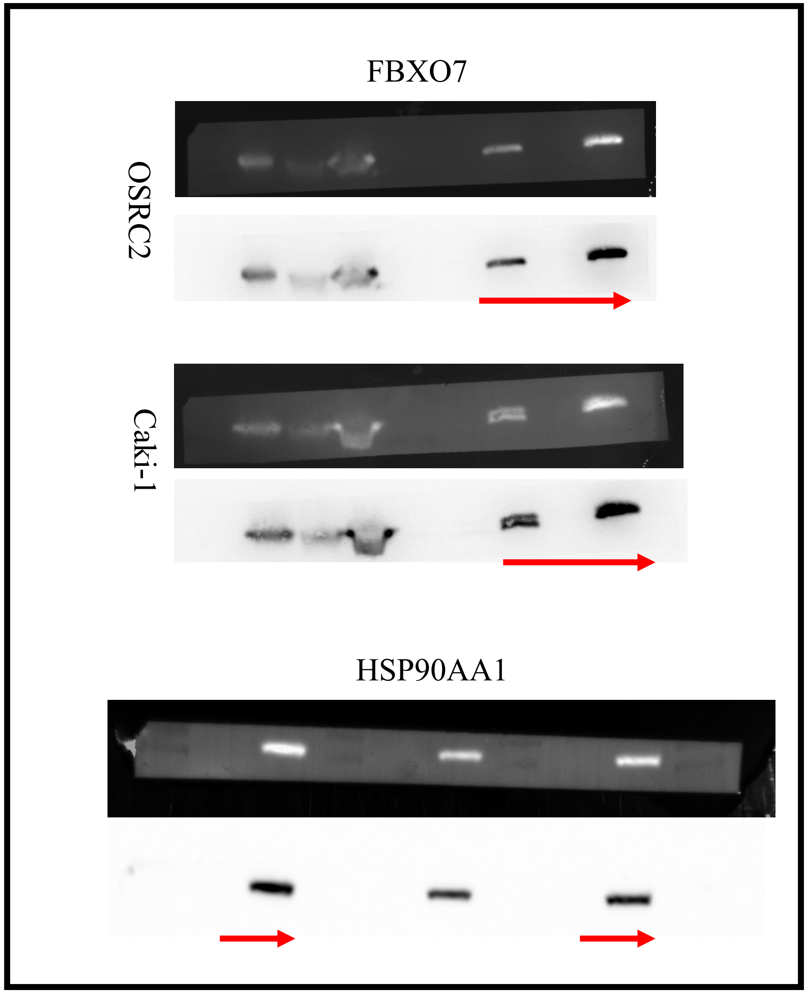
FBXO7
OSRC2
Caki-1
HSP90AA1

Fig. 5C

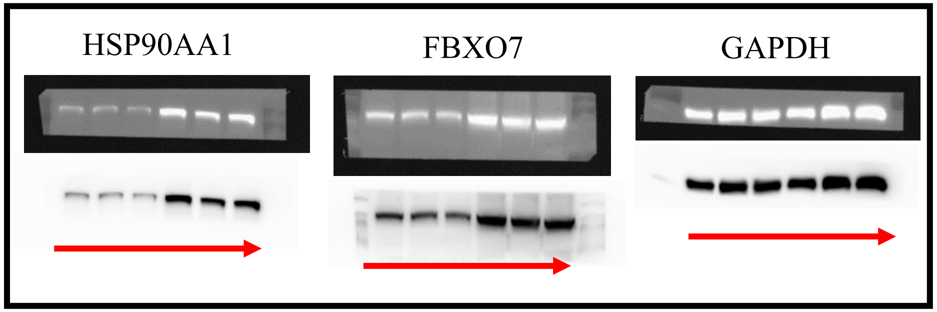


Fig. 5D

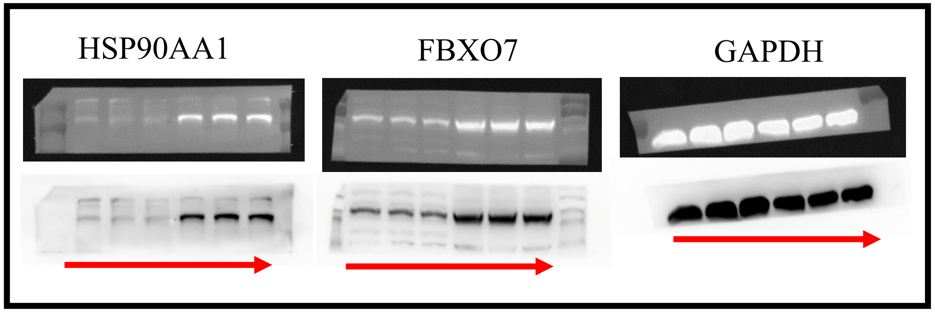


Fig. 5E

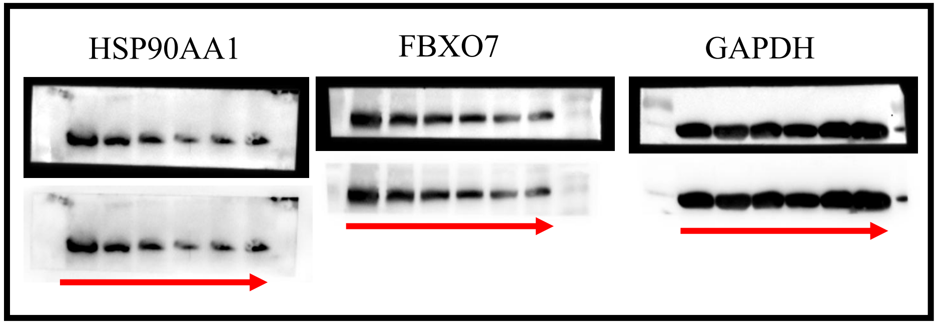


Fig. 5F

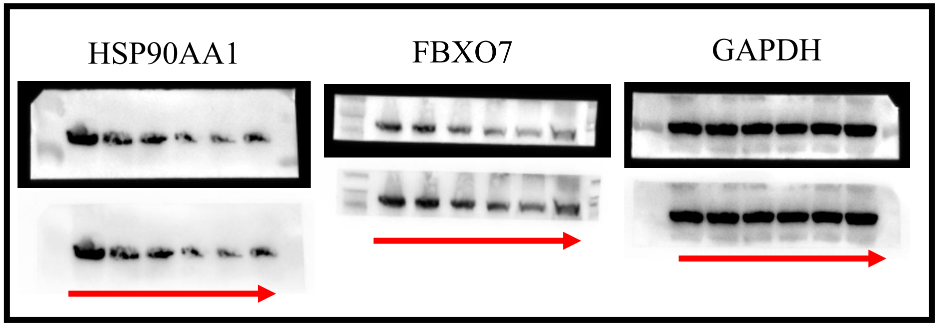


Fig. 5H

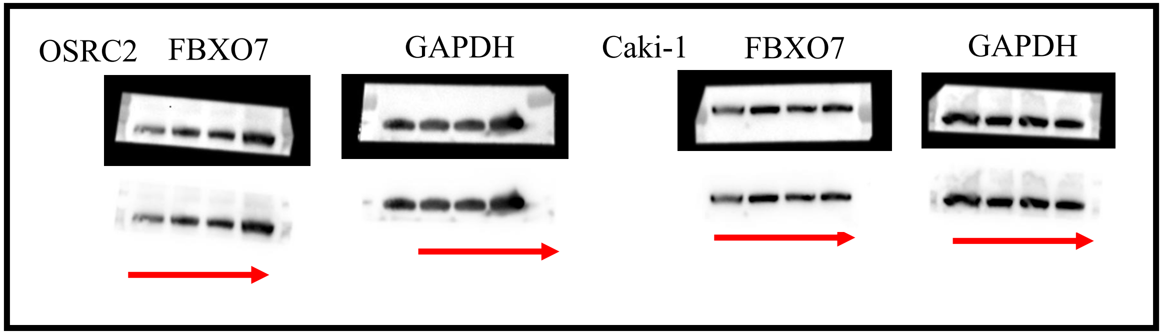

Fig. 7E

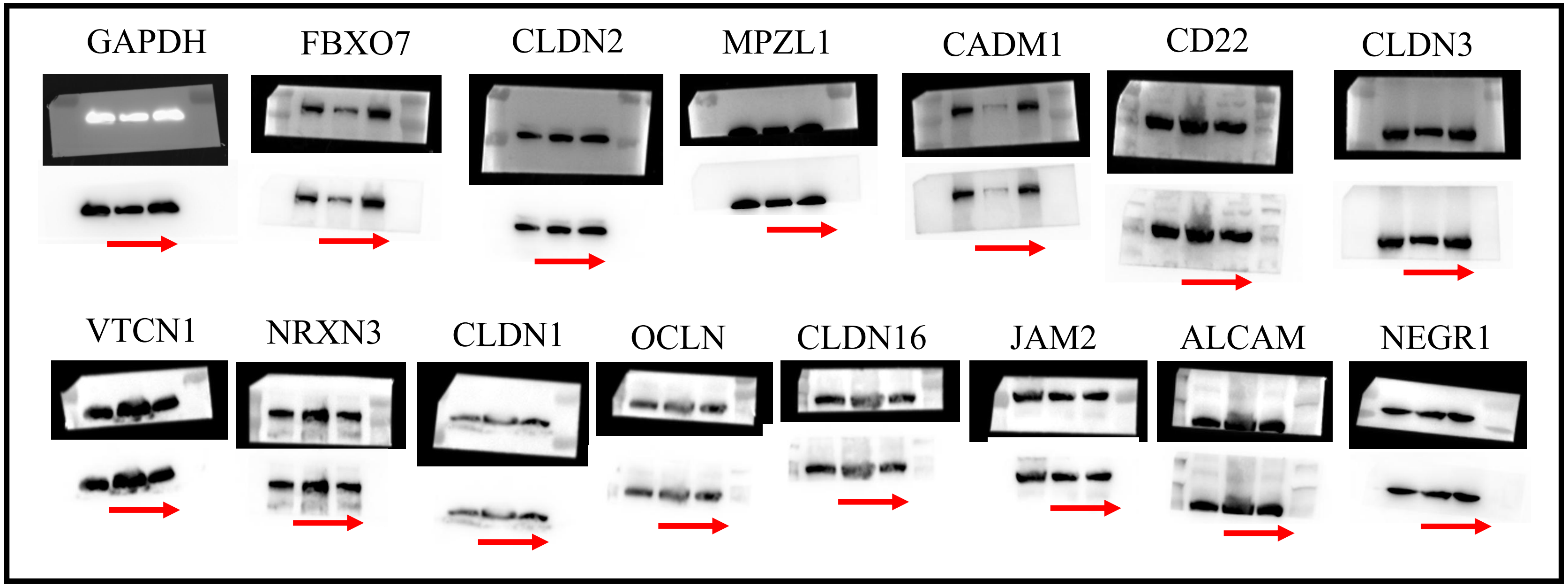
GAPDH
FBXO7
CLDN2
MPZL1
CADM1
CD22
CLDN3
VTCN1
NRXN3
CLDN1
OCLN
CLDN16
JAM2
ALCAM
NEGR1

Fig. 7G

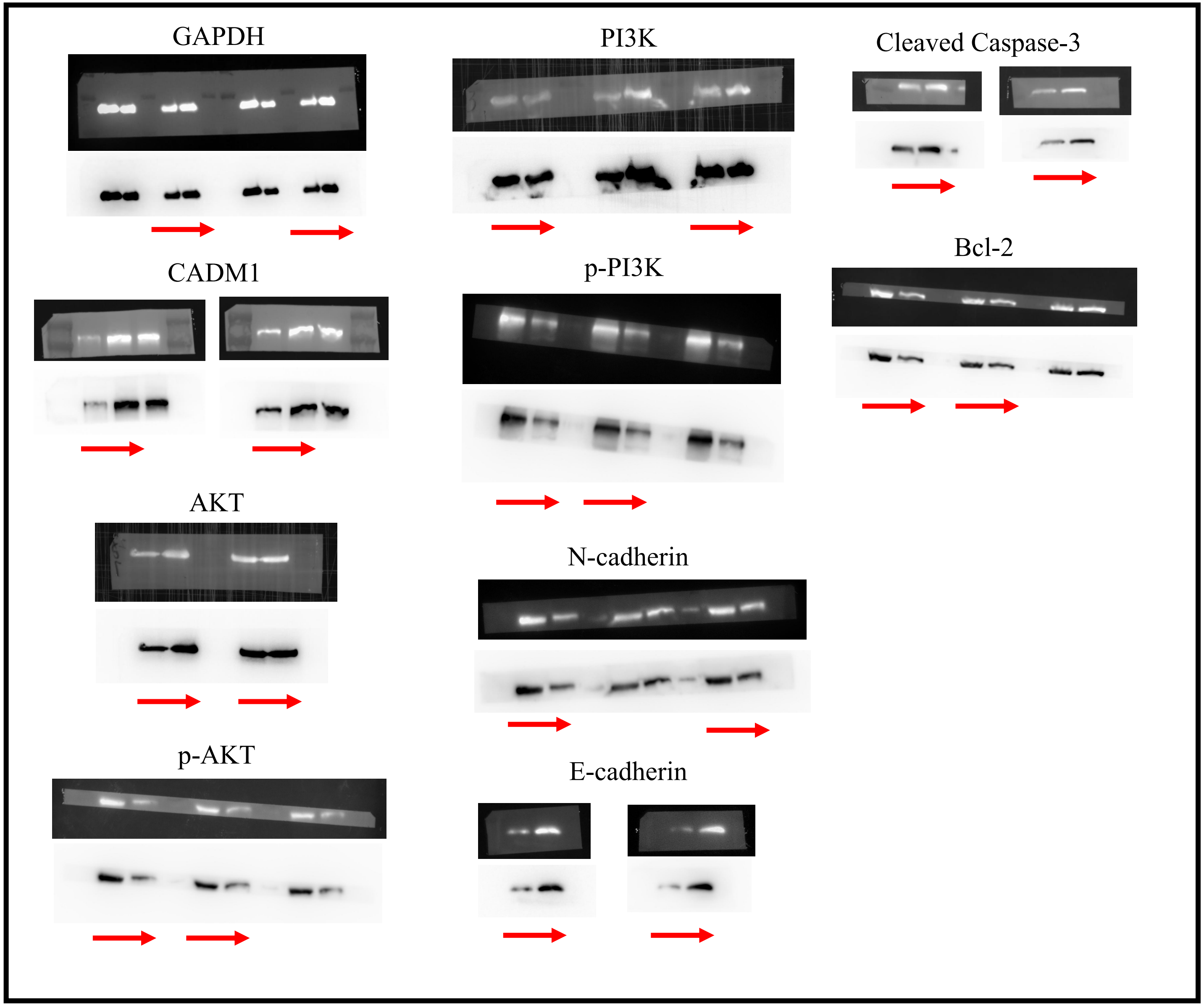
GAPDH
PI3K
Cleaved Caspase-3
CADM1
p-PI3K
Bcl-2
AKT
N-cadherin
p-AKT
E-cadherin

Fig. 7H

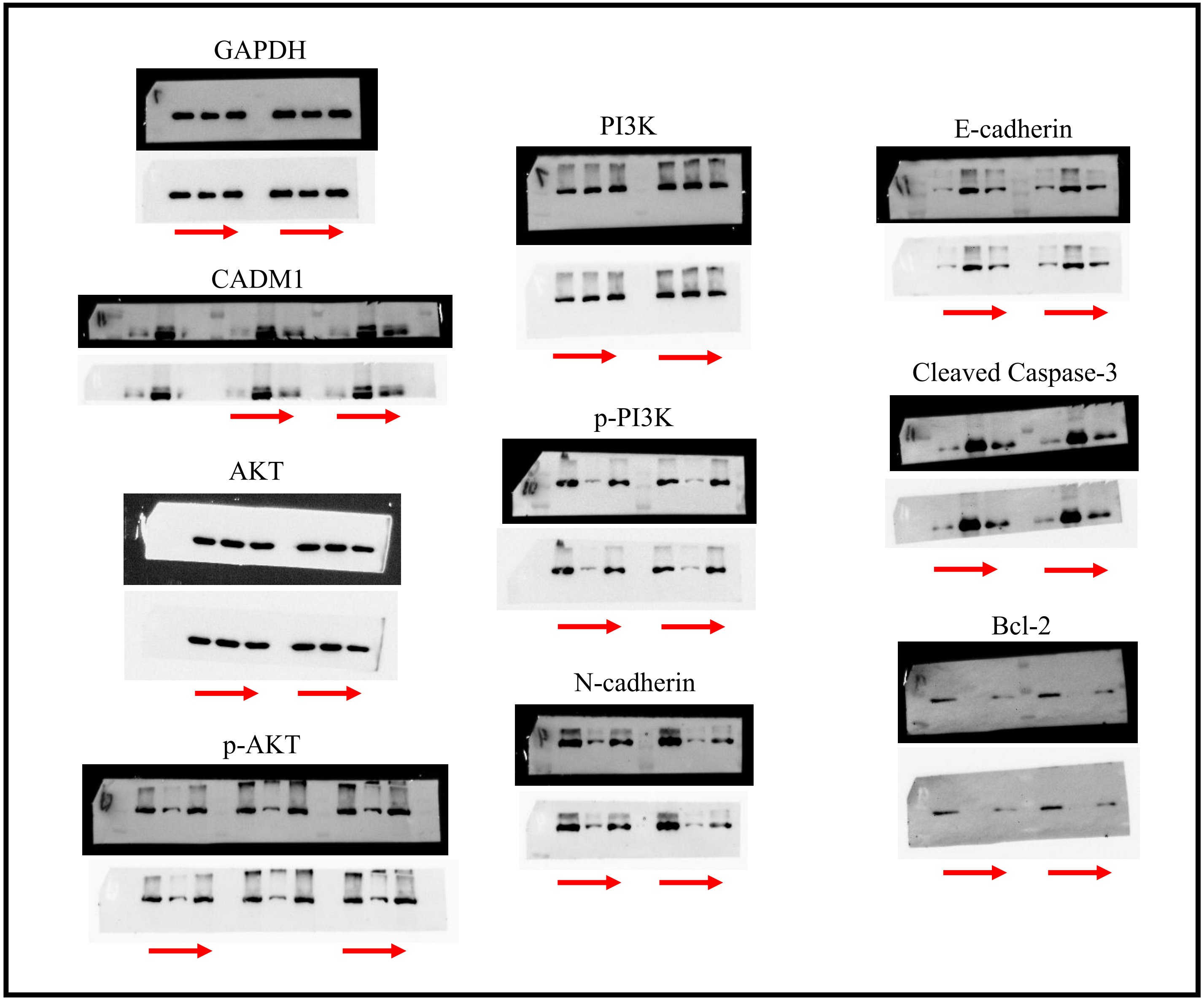
GAPDH
CADM1
AKT
p-AKT
PI3K
p-PI3K
N-cadherin
E-cadherin
Cleaved Caspase-3
Bcl-2
